# Precisely Regulating 2.45 s Pure Blue Room‐Temperature Phosphorescence via Supramolecular Coordination

**DOI:** 10.1002/advs.77398

**Published:** 2026-08-24

**Authors:** Jun Wang, Xingting Que, Zhiming Chen, Jingsheng Zhang, Wei‐Lei Zhou, Wen‐Wen Xu

**Affiliations:** ^1^ School of Chemistry and Materials Science Guizhou Normal University Guiyang People's of Republic China; ^2^ Inner Mongolia Key Laboratory For the Natural Products Chemistry and Functional Molecular Synthesis The Technological Innovation Center of Supramolecular Chinese (Mongolian) Medicine College of Chemistry and Material Science Inner Mongolia Minzu University Tongliao People's of Republic China; ^3^ Department of Thoracic Surgery Xinqiao Hospital Army Medical University (Third Military Medical University) Chongqing People's of Republic China

**Keywords:** blue phosphorescence, energy transfer, precise control, rare earth ions, supramolecular coordination

## Abstract

Achieving tunable blue room‐temperature phosphorescence (BRTP) emission faces forbidden challenges due to difficulties in generating high‐energy triplet excitons and their sensitivity. Herein, a covalently crosslinked purely organic material (BOP@BA) is prepared from boric acid (BA) and 3,5‐dicarboxyphenylboronic acid (BOP) via a simple dehydration reaction. This material exhibits pure deep blue room‐temperature phosphorescence (RTP) with a lifetime of 2.45 s. Unlike individual BA or BOP, dehydrated BOP@BA exhibits a hypsochromic shift to 415 nm, yielding a purely deep‐blue RTP. Meanwhile, the formed covalent skeleton protects the high‐energy triplet excitons from nonradiative deactivation, thereby elongating the lifetime to 2.45 s. Significantly, lanthanide ions (Ln^3+^: Eu^3+^ and Tb^3+^), acting as fine controllers, enable precise tuning of the lifetime through supramolecular coordination and energy transfer, with a precision of as low as 0.1 s and an effective range of 1.71–2.45 s. This work is expected to provide a feasible strategy for constructing ultralong BRTP materials and achieving precise luminescence manipulation.

## Graphical abstract

Herein, a pure deep blue room‐temperature phosphorescence (415 nm) with lifetime up to 2.45 s is successfully achieved by a covalently crosslinking purely organic material based on boric acid (BA) and 3,5‐dicarboxyphenylboronic acid (BOP) through a simple dehydration reaction, which can be adjusted precisely by supramolecular coordination with lanthanide ions (Eu^3+^ and Tb^3+^) acting as fine controllers, with a precision as low as 0.1 s.

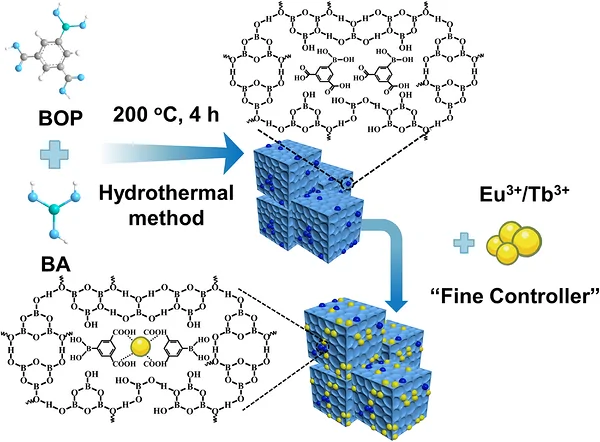

## 1 Introduction

Organic blue phosphorescent materials are a current research hotspot because of their unique color, special luminescent pathway, and long luminescence lifetime, showing broad application prospects in fields of anti‐counterfeiting [1, 2, 3, 4, 5, 6, 7], organic light‐emitting diodes [8, 9, 10, 11, 12], optoelectronic displays [13], and so on [11, 12, 13, 14, 15, 16, 17, 18, 19]. However, conventional molecular design strategies, such as enlarged *π*‐conjugation and supramolecular aggregates, typically lower the excited state energy level, thereby red‐shifting the emission to longer wavelengths [20, 21, 22]. Meanwhile, purely organic phosphorescent systems based on aromatic scaffolds remain largely limited to green and yellow emission bands [23, 24, 25, 26]. Beyond a small number of transition metal‐containing and metal‐organic coordination platforms [27, 28, 29, 30, 31], general organic structures offer few viable pathways for populating triplet excitons that can support efficient blue room‐temperature phosphorescence (BRTP) [7, 32, 33, 34]. Furthermore, the high‐energy triplet excitons underlying BRTP are particularly susceptible to nonradiative quenching via intensified molecular motions and collisions, a drawback far more pronounced than in longer‐wavelength RTP systems [35]. Collectively, these inherent constraints present substantial obstacles to realizing efficient, long‐lived BRTP, severely restricting the further development and practical deployment of high‐performance blue organic phosphorescent materials.

In recent years, notable progress has been made in the development of organic blue phosphorescent materials [34, 36, 37, 38, 39, 40, 41, 42, 43]. For instance, Ma et al. developed efficient dark BRTP materials by doping methyl benzoate derivatives into poly(vinyl alcohol) matrices, achieving ultrabroadly tunable phosphorescence lifetimes ranging from 32.8 to 1925.8 ms [34]. Controlling the aggregation states of chromophores (e.g., anionic cellulose trimellitates) has also been demonstrated as an effective strategy for blue‐emitting RTP, yielding color‐tunable phosphorescence with strong excitation‐dependent characteristics [22]. Additionally, pure hydrocarbon materials, used as hosts, have shown promising applications in blue organic light‐emitting diodes (OLEDs) when combined with phosphorescence‐sensitized multi‐resonance thermally activated delayed fluorescence [12]. Recently, narrowband blue RTP materials based on indolo[3,2,1‐kl]phenothiazine derivatives have been reported, opening new opportunities for advanced optoelectronic sensing and display applications [37]. Despite these remarkable advances, the rational fabrication of long‐lived BRTP materials with lifetimes exceeding 2 s, as well as their precise and reliable lifetime tuning, remains largely underexplored.

**SCHEME 1:**
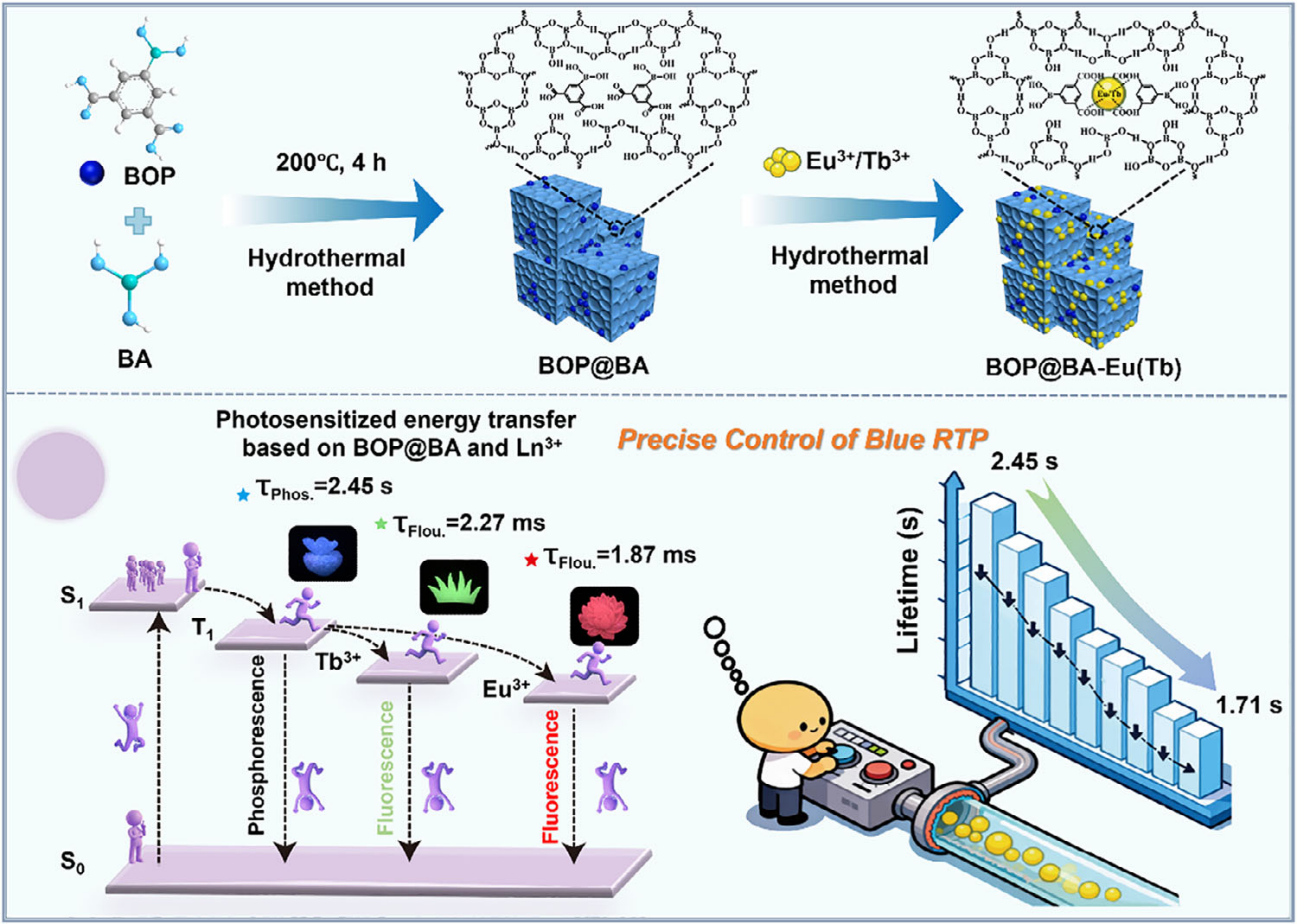
Schematic illustration of precisely regulating 2.45 s pure blue RTP with Ln^3+^ as a fine controller.

In this work, a purely organic BRTP material (BOP@BA) with a lifetime of 2.45 s is successfully prepared by simple calcination of boric acid (BA) and 3,5‐dicarboxyphenylboronic acid (BOP) in a simple drying oven, without any further processing (Scheme 1). Compared with individual BOP or BA, the calcinated BOP@BA product exhibits a higher triplet state, which corresponds to blue emission. Dehydrated BA (DBA) and abundant hydrogen bonds form a rigid skeleton that stabilizes triplet excitons, inducing an ultralong lifetime of 2.45 s. Significantly, the introduction of lanthanide ions (Eu^3+^ and Tb^3+^) enables precise modulation of the blue phosphorescence lifetime through intermolecular energy transfer between the organic phosphor and lanthanide centers. The tuning precision can be well controlled within 0.1 s, thereby allowing selective and reliable access to phosphorescence lifetimes ranging from 1.71 to 2.45 s. Furthermore, the synthetic versatility and elimination of complex post‐treatment procedures markedly simplify the fabrication process, rendering this material highly promising for scalable practical applications.

## 2 Results and Discussion

**FIGURE 1:**
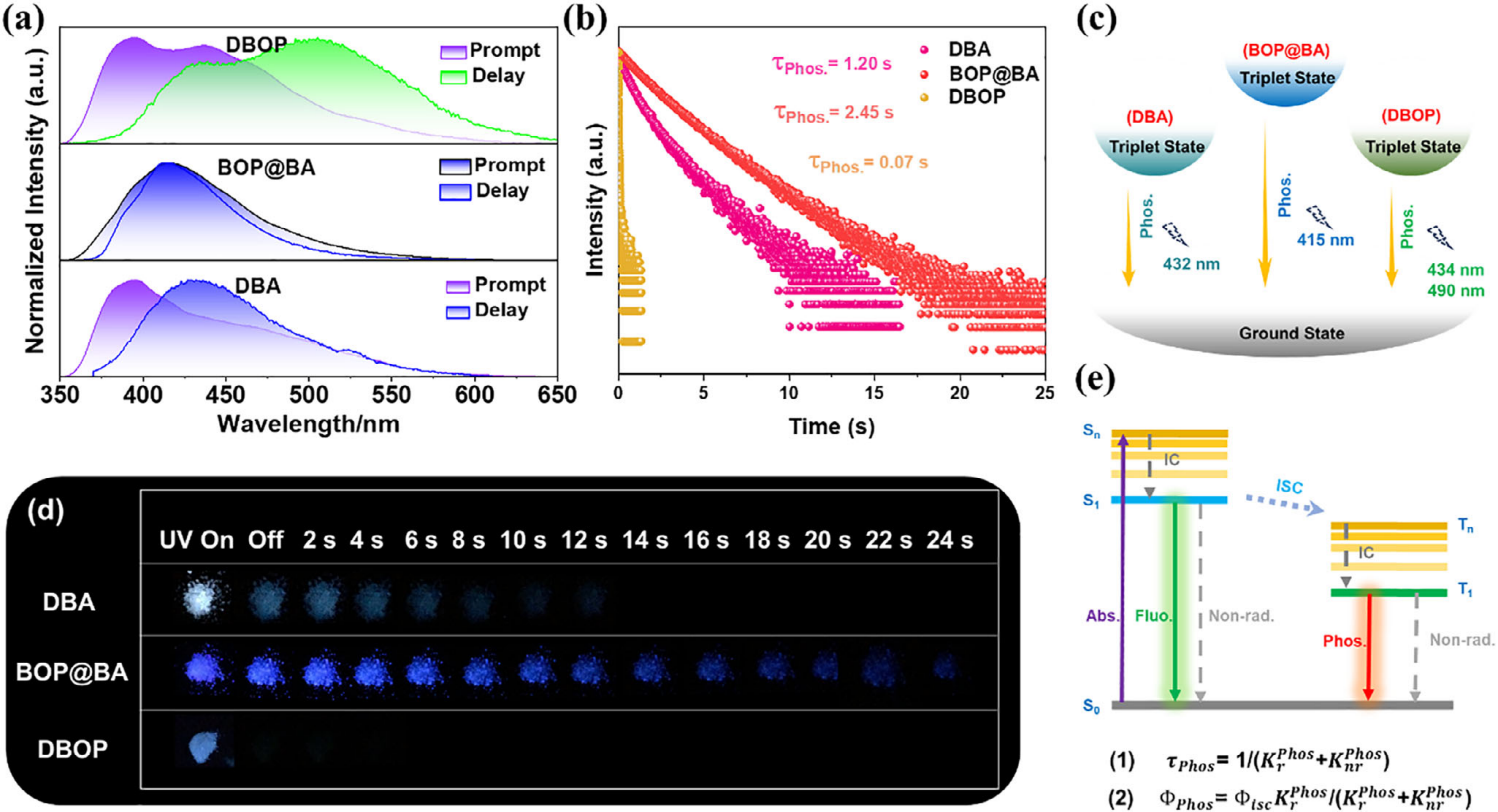
(a) Prompt, and delayed spectra of DBA, DBOP, and BOP@BA in solid state under ambient environment; (b) Time‐resolved photoluminescence decay plot of DBA (at 432 nm), DBOP (at 505 nm), and BOP@BA (at 415 nm); (c) Change in emission energy level of DBA, DBOP, and BOP@BA; (d) Photographs of DBA, DBOP, and BOP@BA before and after ceasing 254 nm UV light; (e) Jablonski diagram and the equation of phosphorescence lifetime (τ*_Phos_*) and phosphorescence quantum yield (Φ*_Phos_*).

Three dehydrated solid materials (DBA, DBOP, BOP@BA) were fabricated by thermal dehydration of their corresponding pristine aqueous precursor solutions (BA, BOP, and a mixture of BA and BOP) in a convection oven at 200°C for 4 h. According to steady‐state and delayed photoluminescence spectra, individual DBA and DBOP exhibited distinct delayed emission bands centered at 432, 434, and 505 nm, respectively, while maintaining an identical prompt emission at 395 nm (Figure 1a, Figure S1a, and Table S1). The longer emission wavelengths and millisecond‐to‐second photoluminescence lifetimes (1.20 s for DBA and 0.07 s for DBOP) unambiguously confirmed that the bands at 432 and 505 nm originated from triplet‐involved phosphorescence, with phosphorescence quantum yields of 7.89% (DBA) and 10.7% (DBOP) (Figure 1b and Table S1).

Notably, upon joint dehydration of BA and BOP to form the covalent complex BOP@BA, both prompt fluorescence and phosphorescence converged into a characteristic single emission at 415 nm, giving rise to intense deep blue luminescence (Figure 1a,c, Figure S1b, and Table S1). To verify the nature of the emerging emission, a time‐gated detection protocol (delay ≥ 0.1 ms) was employed. The results confirmed that the emission centered at 415 nm exhibited a distinct long‐lived characteristic, with a lifetime exceeding 2 s and a quantum yield of 16.2% (Figure 1b and Table S1). Furthermore, the lifetime of the composite was strongly dependent on the BA/BOP molar ratio employed during thermal dehydration. As the BOP molar fraction increased from 0% to 20%, the lifetime first increased and then decreased (Figure S2). A maximum lifetime of 2.45 s was achieved at a BOP ratio of 5%, accompanied by a durable blue afterglow that persisted for over 24 s (Figure 1d). Based on these observations, the new emission band and lifetime enhancement are reasonably attributed to the covalently crosslinked product formed at elevated temperatures. Specifically, thermal treatment drove BA and BOP to form a dehydrated covalent crosslinking network with a specific surface area of 3.18 m^2^ g^−1^ and an average pore size of 10.21 nm (Figure S3a), which possessed a stronger, more rigid structure and improved thermal stability compared to DBA and DBOP. This structural rigidity efficiently suppressed nonradiative deactivation, including molecular vibration and rotation, intermolecular collisions, and oxygen‐induced quenching of triplet excitons, thereby prolonging the lifetime (Figure 1e).

**FIGURE 2:**
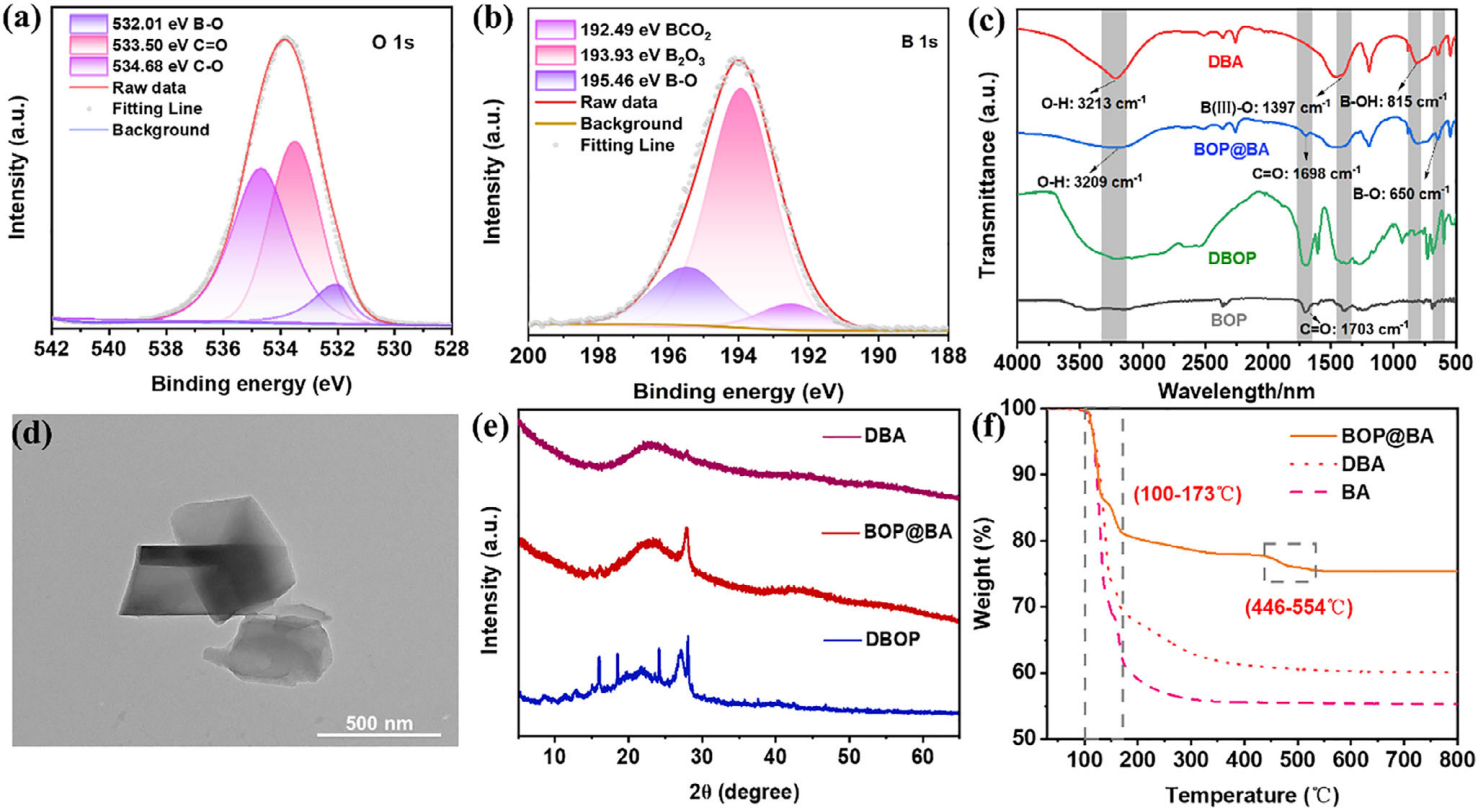
High‐resolution XPS spectra of BOP@BA: (a) O 1s and (b) B 1s; (c) FT‐IR spectroscopy of DBA, BOP, DBOP, and BOP@BA; (d) TEM image of BOP@BA; (e) XRD patterns of DBOP, DBA, and BOP@BA; (f) TGA analysis of BA, DBA, and BOP@BA.

To confirm this assumption, X‐ray photoelectron spectroscopy (XPS), Fourier transform infrared (FT‐IR) spectroscopy, X‐Ray diffraction (XRD), scanning electron microscopy (SEM), transmission electron microscopy (TEM), and thermogravimetric analysis (TGA) were performed. XPS survey confirmed that the addition of BOP during calcination results in elevated intensities of both oxygen and boron signals, verifying the successful introduction of BOP into the BA matrix (Figure 2a,b, and Figure S3b). The high‐resolution B 1s spectrum exhibited three distinct peaks centered at 192.49, 193.93, and 195.46 eV, assignable to BCO_2_, B_2_O_3_, and B─O moieties, respectively (Figure 2b). In the O 1s spectrum, three characteristic peaks appeared at 532.01, 533.50, and 534.68 eV, corresponding to B─O, C═O, and C─O bonds, respectively (Figure 2a).

Corroboratively, FT‐IR spectra revealed characteristic vibrations associated with B─O covalent bonds and hydroxyl groups (Figure 2c). The bands at 650 and 815 cm^−1^ corresponded to B─O and B─OH stretching vibrations, while the peak at 1397 cm^−1^ was assigned to B(III)‐O stretching. The broad absorption region at 3200–3420 cm^−1^ corresponded to O─H stretching vibrations, implying that abundant hydroxyl groups remained despite high‐temperature calcination. SEM images revealed that, compared to DBA, the composite BOP@BA adopts a sheet‑stacked morphology and a smoother surface, with no carbon dot‑like species formed (Figure S4). TEM images further verified the sheet‑like structure with a typical lateral dimension of approximately 500 nm (Figure 2d).

According to the XRD patterns, the calcined product DBA exhibited a broad diffraction feature characteristic of an amorphous polymer (Figure 2e). Meanwhile, high‑temperature calcination altered the crystalline structure of pristine BOP (reflections at 8.05°, 12.28°, 16.22°, 21.48°, 27.47°). Calcined DBOP showed diffraction peaks at 15.99°, 18.46°, 24.14°, 27.08°, and 28.05°, and the characteristic reflection at 27.88° observed in BOP@BA indicated the retention of crystalline ordering. Collectively, these results demonstrate that thermal treatment facilitates the dehydration polymerization between BA and BOP and the formation of a covalently crosslinked polymeric network.

TGA was employed to characterize thermal dehydration behavior and structural stability. All samples, including pristine BA, DBA, and BOP@BA, exhibited significant weight loss within the temperature range of 100°C–173°C (Figure 2f). Notably, after the first calcination step, the weight loss of DBA and BOP@BA was considerably suppressed, with BOP@BA displaying the lowest mass loss among the three samples. Upon further heating to 800°C, the TGA curves of BA and DBA remained nearly unchanged, whereas BOP@BA showed a slight weight loss between 446°C and 554°C, attributed to the gradual thermal decomposition of the BOP moiety. These observations confirm that thermal treatment at ≥100°C effectively triggers dehydration, and the incorporation of BOP further facilitates this process, thereby generating a more robust covalent crosslinking skeleton.

The participation of BOP in the formation of the framework was further verified by FT‐IR spectroscopy (Figure 2c). The characteristic carbonyl stretching peak shifted from 1703 cm^−1^ in pure BOP to 1698 cm^−1^ in BOP@BA, supporting the formation of covalent linkages during dehydration. Accordingly, the synergistic calcination treatment of BOP and BA successfully stabilizes the material by constructing a rigid covalent crosslinking structure. Such a rigid structure suppresses nonradiative deactivation of excited states by strengthening intermolecular interactions and improving structural ordering, thereby providing a critical structural prerequisite for achieving ultralong and stable RTP.

The effects of calcination temperature on the structure and phosphorescence of BOP@BA were further investigated. The experimental results revealed that as the temperature increased from 140°C to 200°C, the originally negligible phosphorescence peak at 140°C became an obvious emission at 415 nm at 160°C. It was further enhanced from 160°C to 200°C (Figure S5a). As the temperature was increased to 220°C, this enhancement declined, suggesting that 200°C was the optimal calcination temperature for generating the covalently crosslinked structure and BRTP. Meanwhile, XRD patterns demonstrated a corresponding transformation from a crystalline structure (140°C) to an amorphous structure (≥160°C) (Figure S5b). The agreement between the photophysical and structural changes indicated that the amorphous crosslinked structure was closely related to the robust phosphorescence behavior. Owing to the hydrophilic boronic acid moieties, BOP@BA exhibited a slight reduction in phosphorescence lifetime when exposed to ambient air over 36 h, while it maintained robust photostability under continuous UV irradiation for 3 h (Figure S6).

To elucidate the origin of the deep blue and long‐lived phosphorescence, steady‐state and time‐resolved spectral characterizations were performed on DBA, BOP, DBOP, and BOP@BA, and the nature of the long‐lived emissive species was systematically analyzed. As revealed by the above results, pristine DBA exhibited a single delayed‐emission centered at 432 nm, whereas DBOP displayed two distinct delayed‐emission peaks at 434 and 505 nm (Figure 1a). Similarly, BOP emitted dual delayed‐emission bands at 417 and 498 nm (Figure S7). Together, the dual delayed emissions, excitation‐dependent luminescence behavior of BOP and DBOP, as well as the high‐temperature dehydration process, suggested two plausible origins for the enhanced BRTP: (1) BOP harbors two long‐lived emissive species at ambient conditions, corresponding to the monomeric state and aggregated state, respectively; (2) high‐temperature calcination gives rise to a new type of luminescent species responsible for the long‐lived blue emission.

To verify the first hypothesis, a dilute BOP solution (2 × 10^−7^ m) was prepared to disrupt intermolecular *π–π* stacking and aggregate formation, and its delayed luminescence spectrum was recorded at 77 K (Figure S8). The result showed that the BOP solution still exhibited a dominant delayed emission at around 478 nm with no blue emission observed, thus excluding the possibility of a single‐molecule state in the first hypothesis. To demonstrate the second hypothesis, solid BOP was ground with BA, and the resulting mixture (denoted BOP+BA, 5 wt.%) was subjected to two distinct drying protocols: lyophilization (which supplies no thermal energy for dehydration) and heating at 65°C (which provides sufficient thermal energy to drive dehydration). As evidenced by the delayed luminescence spectra, lyophilized BOP+BA displayed emission profiles (λ > 500 nm) analogous to those of neat BOP solid and BOP solution measured at 77 K (Figure S9). In sharp contrast, heating at 65°C induced a pronounced enhancement of the delayed blue emission for BOP+BA, confirming that elevated temperature promotes the hypsochromic luminescence in the BOP+BA system. These results, combined with the occurrence of dehydration after thermal treatment, confirm that the intense deep blue phosphorescence of BOP@BA primarily originates from new emissive species generated by the dehydrated coupling product of BOP and BA. Conventional aggregates stabilize triplet excitons via intermolecular π‐orbital delocalization. This delocalization lowers the intrinsic T_1_ energy level and induces red‐shifted emission. In contrast, calcination reconstructs BOP structures, generating hypsochromic emission. The rigid crosslinked matrix stabilizes triplet excitons, extends lifetimes, and enables ultralong BRTP.

**FIGURE 3:**
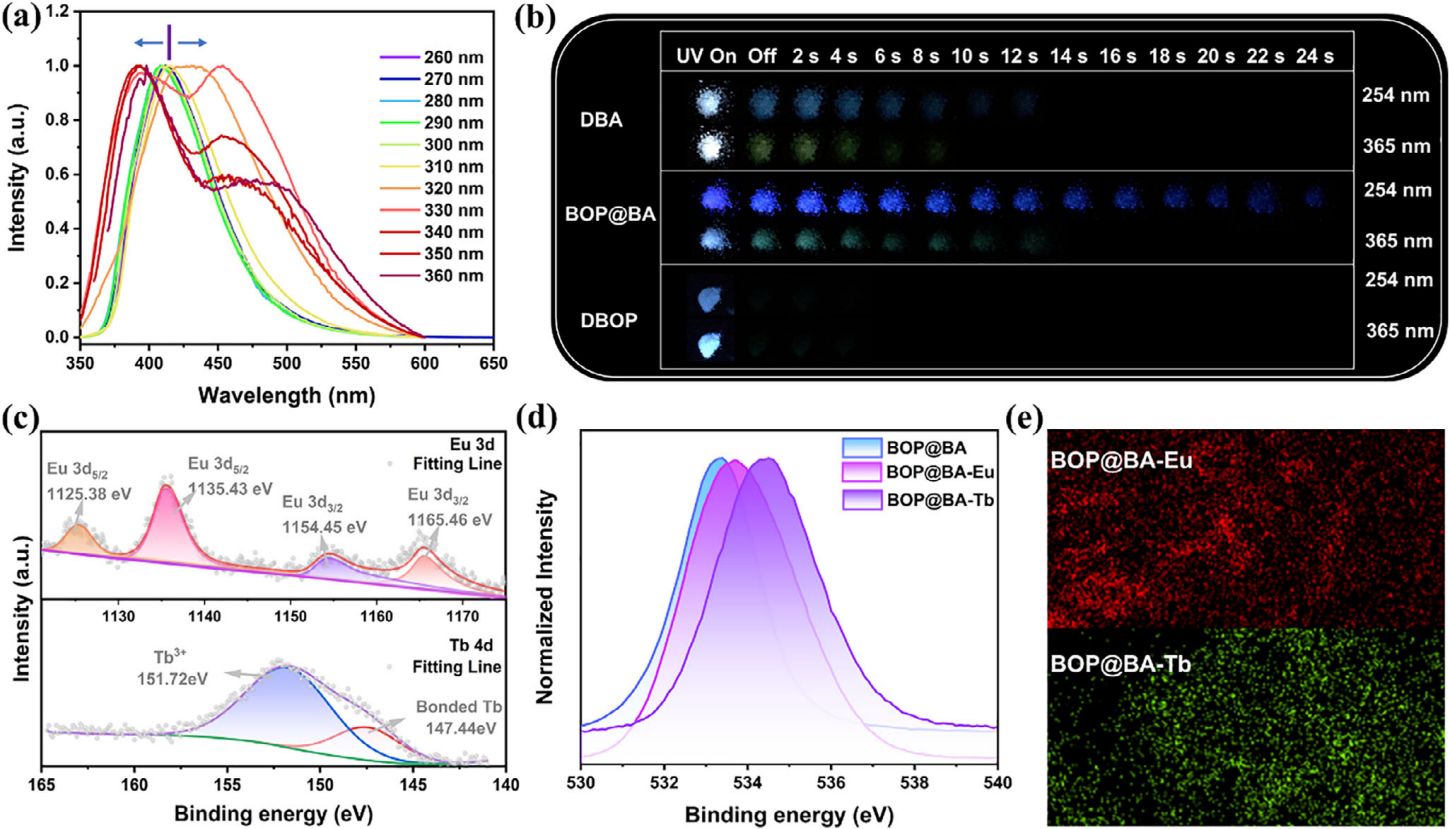
(a) Excitation‐dependent delayed emission of BOP@BA with changing excitation wavelength from 260 to 360 nm; (b) Photographs of DBA, BOP@BA, and DBOP before and after ceasing 254 nm or 365 nm ultraviolet radiation; XPS spectra of (c) and (d) of BOP@BA, BOP@BA‐Eu, and BOP@BA‐Tb; (e) EDS spectra of BOP@BA‐Eu and BOP@BA‐Tb.

Nonetheless, the reversible nature of the dehydration reaction leaves a fraction of unreacted BOP. When the excitation wavelength was tuned from 260 to 360 nm, the single emission peak at 415 nm resolved into two distinct bands centered at 394 nm and 453–478 nm, resembling the dual emission characteristics of BOP and DBOP. Accordingly, BOP@BA exhibits evident excitation‐dependent emission and multicolor afterglow (Figure 3a,b). Unlike BOP@BA, the multicolor afterglow observed for DBA arises mainly from red shifted emission, as reported previously [7]. To rule out delayed fluorescence and thermally activated delayed fluorescence (TADF), temperature‐dependent photoluminescence measurements were recorded on solid BOP@BA. The 415 nm emission intensity decreased progressively with increasing temperature, and no characteristic TADF trend was detected, unambiguously verifying the phosphorescent nature of this emission (Figure S10).

As a fundamental primary color for optoelectronic devices, deep blue phosphorescence holds substantial promise for applications in OLED displays and advanced photoelectric materials. The development of readily tunable deep‐blue phosphorescent systems is therefore critical to advancing the design and practical deployment of high‐performance blue‐emitting materials. Motivated by the isophthalic acid motif in BOP and the strong coordination propensity of carboxylate groups toward lanthanide ions (Ln^3+^), we hypothesized that metal–organic coordination could serve as an effective strategy to modulate phosphorescence properties. Accordingly, Eu^3+^ and Tb^3+^ were selected as representative Ln^3+^ ions and incorporated into BOP@BA during thermal annealing. XPS and EDS analyses confirmed the successful introduction of Eu^3+^ and Tb^3+^ and homogeneous distribution within the BOP@BA matrix (Figure 3c,e). Furthermore, high‐resolution XPS spectra in the O 1s region showed significant shifts in binding energy upon the introduction of Eu^3+^ and Tb^3+^, providing direct evidence for coordination interactions between oxygen‐donor sites and Ln^3+^ ions (Figure 3c). Consistent with these observations, IR spectra revealed attenuation of the characteristic carboxylate group vibrations in BOP@BA‐Eu and BOP@BA‐Tb, which further verifies the formation of Ln^3+^‐carboxylate coordination bonds (Figure S11).

**FIGURE 4:**
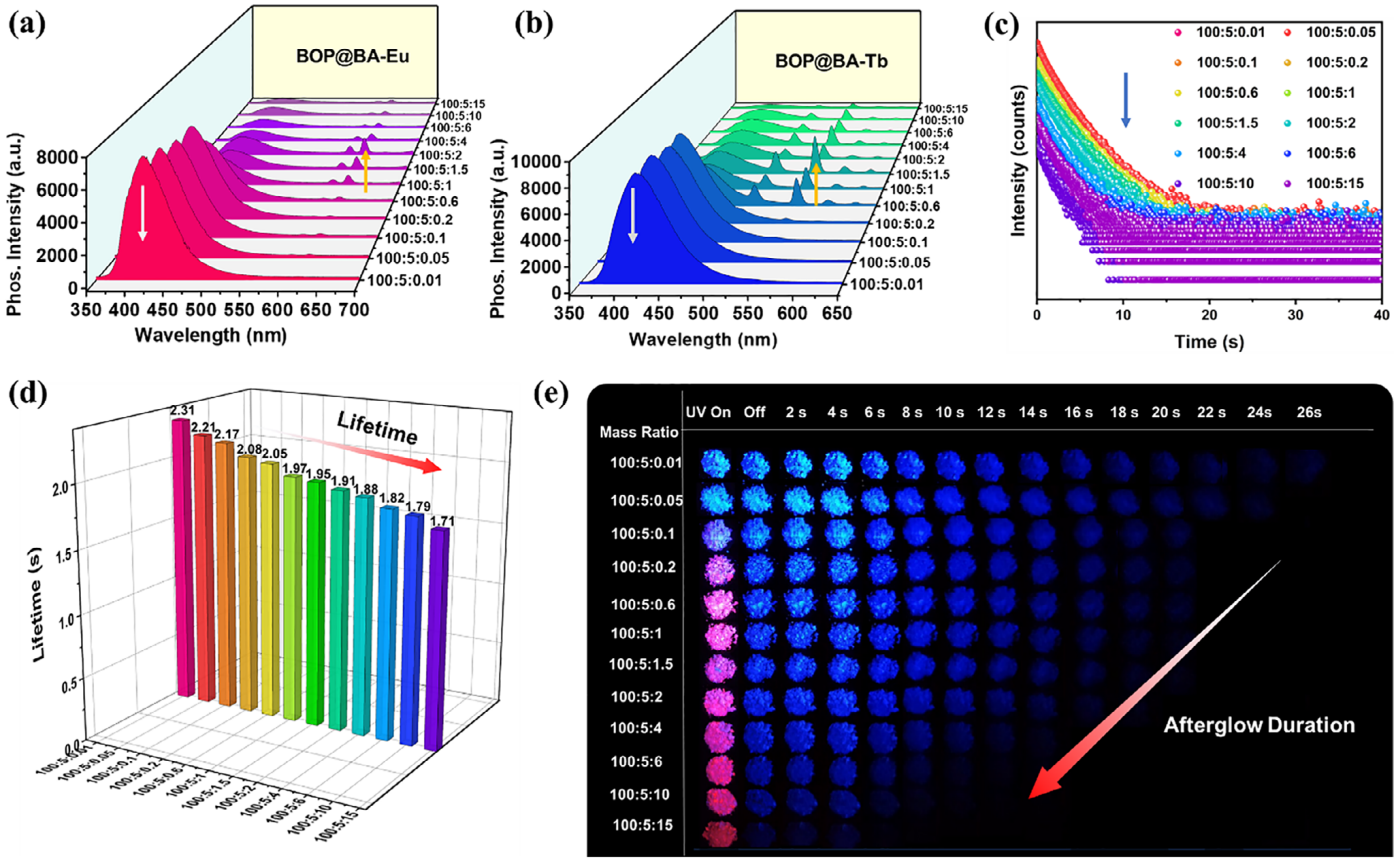
Delay spectra of (a) BOP@BA‐Eu with gradually increasing the mass ratio of Eu^3+^ and (b) BOP@BA‐Tb with gradually increasing the mass ratio of Tb^3+^ under ambient environment (excitation wavelength: 265 nm); (c) Time‐resolved luminescence decay plots and (d) averaged lifetimes of BOP@BA‐Eu at 415 nm with gradually increasing the mass ratio of Eu^3+^; (e) Afterglow photographs of BOP@BA‐Eu with gradually increasing the mass ratio of Eu^3+^ before and after ceasing 254 nm UV light.

Spectral characterization confirmed that the incorporation of Eu^3+^ and Tb^3+^ had negligible influence on the emission energy level of BOP@BA, as evidenced by the unchanged emission wavelength at 415 nm and consistent blue afterglow (Figure S12). As the doping ratio of Ln^3+^ increased from 0 to 15 wt.%, the phosphorescence intensity of BOP@BA decreased gradually and continuously (Figure 4a,b, and Figure S13). Meanwhile, the characteristic emission peaks of Ln^3+^ emerged and progressively intensified. In line with the typical photophysical behavior of luminescent donor‐acceptor pairs, the simultaneous decay of donor phosphorescence (BOP@BA) and emergence/enhancement of acceptor emission (Ln^3+^) strongly indicated energy transfer (ET) from BOP@BA to Ln^3+^. The gradual elevation of ET efficiencies from 5.71% to 30.2% for BOP@BA‐Eu and from 6.12% to 28.98% for BOP@BA‐Tb, in good accordance with the Ln^3+^ doping ratio, further verified that the introduction of Eu^3+^ and Tb^3+^ effectively promoted the ET process (Tables S2 and S3).

Intriguingly, the introduction of Ln^3+^ not only modulates phosphorescence intensity but also precisely regulates the phosphorescence lifetime and afterglow duration. Taking Eu^3+^ as an example, the incorporation of 0.01 wt.% Eu^3+^ induced a subtle reduction in the phosphorescence lifetime of BOP@BA from 2.45 to 2.31 s (Figure 4c,d). To exclude potential instrumental deviations, the doped Eu^3+^ was further increased to 0.05 wt.%, which led to a gradual decline in lifetime to 2.21 s. This tendency remained unchanged, and the lifetime further decreased to 2.17 s as the Eu^3+^ doping ratio reached 0.1 wt.%. Such stepwise attenuation strongly suggests a systematic regulation effect rather than random fluctuation.

A series of BOP@BA‐Eu samples with varying Eu^3+^ loadings was then synthesized and systematically characterized. By analyzing the correlation between phosphorescence lifetime and Eu^3+^ doping ratio, we observed that the lifetime of BOP@BA decreases monotonically from 2.45 to 1.71 s as the Eu^3+^ content increases from 0 to 15 wt.%. Notably, the interval between any two adjacent lifetime values was less than 0.1 s (control precision < 5%), with most differences confined within 0.05 s, demonstrating excellent controllability.

To the best of our knowledge, blue phosphorescence modulation with such high precision remains exceptionally scarce among reported RTP materials. This precise lifetime control manifests most intuitively in the afterglow behavior of BOP@BA‐Eu. With increasing molar fraction of Eu^3+^, the afterglow duration of successive samples exhibited subtle yet distinguishable variations (Figure 4e, Figure S14), consistent with the incremental changes in phosphorescence lifetime. Although Eu^3+^ incorporation endows BOP@BA with distinct luminescence colors under 254 and 365 nm excitation, the composite BOP@BA‐Eu^3+^ retained efficient deep blue persistent RTP, which benefits from the millisecond‐scale excited‐state lifetimes of Eu^3+^ centers. Substitution of Eu^3+^ with Tb^3+^ yielded analogous evolutions in phosphorescence lifetime and afterglow profiles (Figures S15–S17), validating the generality of this supramolecular strategy for finely controllable deep blue RTP using Ln^3+^ ions as “precision tuning motifs”.

**FIGURE 5:**
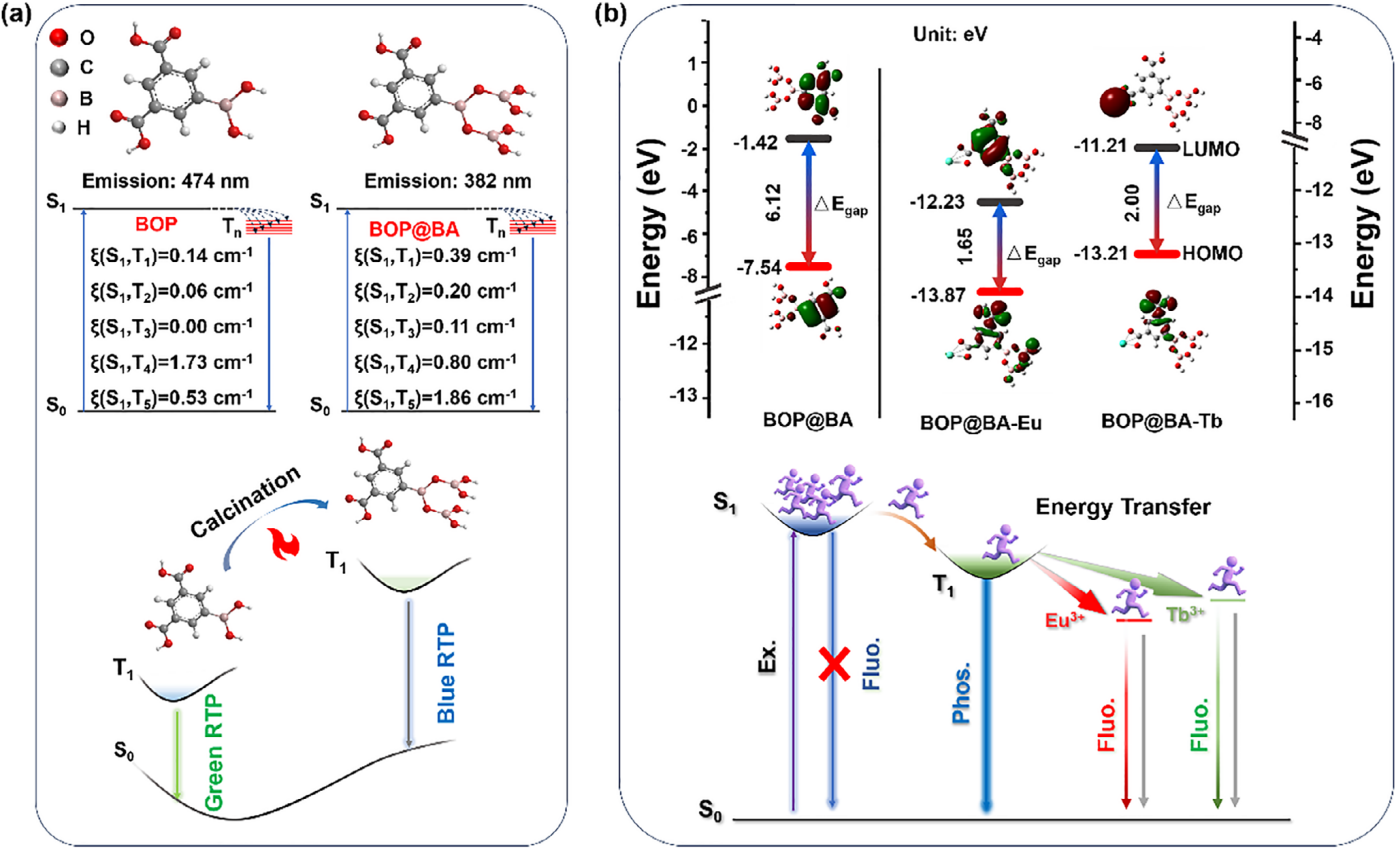
The theoretical calculation through simplified model molecules. (a) The optimized structures (top), possible ISC channels and their ξ values (middle), and schematic illustration of change in T_1_ energy level before and after calcination (down); (b) ΔE_gap_ of BOP@BA, BOP@BA‐Eu, BOP@BA‐Tb (top), and schematic illustration of the ET process (down).

Time‐dependent density functional theory (TDDFT) calculations were further performed to demonstrate the intrinsic origin of the BRTP and ET process [44]. Using BOP as the reference model, theoretical analysis indicated that the ISC process in BOP occurred primarily from the lowest singlet excited state (S_1_) to the triplet excited states T_4_ and T_5_, with corresponding spin–orbit coupling (SOC) matrix elements (ξ) of 1.73 and 0.53 cm^−^^1^, respectively (Figure 5a). To simulate the luminescent center of BOP@BA, BA was introduced to model the dehydrated structure. Notably, the two major ISC pathways remained, with the SOC matrix elements of 0.80 and 1.86 cm^−1^. Meanwhile, the ξ values of the other three ISC pathways (S_1_→T_1_: 0.39 cm^−1^, S_1_→T_2_: 0.20 cm^−1^, and S_1_→T_3_: 0.11 cm^−1^) were significantly higher. Compared with BOP, BOP@BA exhibited more ISC pathways and larger ξ values, enabling more efficient singlet‐to‐triplet exciton conversion. Consequently, the population of singlet excitons decreased accordingly, while the density of triplet excitons increased. This accounts for the characteristic pure BRTP of BOP@BA and the spectral overlap between prompt and delayed emission profiles. Furthermore, thermal dehydration drastically reshaped the excited‐state energy levels responsible for luminescence. The emission wavelength of the dehydrated structure (382 nm for BOP@BA) exhibited a significant hypsochromic shift relative to that of the BOP monomer (474 nm), a trend consistent with experimental observations. These results further indicate that BOP structural change caused by high‐temperature dehydration triggers the blue shift in emission and the characteristic BRTP, together with high‐performance ISC efficiency. The slight discrepancy between theoretical calculations and experimental results can be attributed to the distinct local luminescent environments of the ideal model and the real covalent network.

Furthermore, the energy gaps (ΔE_gap_) were quantitatively evaluated to elucidate the ET process between BOP@BA and Ln^3+^. Theoretical results revealed that the HOMO and LUMO levels of BOP@BA were located above −8 eV, whereas those of BOP@BA‐Ln complexes shifted to below −8 eV (Figure 5b). Notably, the energy gap of BOP@BA was calculated to be 6.12 eV, which decreased sharply to 1.65 eV for BOP@BA‐Eu and 2.00 eV for BOP@BA‐Tb upon coordination. Such a reduction in the energy gap directly accounts for the luminescence color conversion from deep blue (BOP@BA) to green (BOP@BA‐Tb) and red (BOP@BA‐Eu). Meanwhile, the donor‐acceptor distance was calculated to be 0.56 nm (Figure S18). According to a previous report, a distance of 1–10 nm is a critical prerequisite for efficient resonance ET [45], and a much shorter distance of 0.56 nm did not support phosphorescence resonance ET. Combined with the appearance of charge transfer signals and the gradual decline in phosphorescence quantum yields as Ln^3+^ doping content increased (Tables S2 and S3, and Figure S19), these results hinted that the dominant mechanism responsible for precise lifetime tuning was the classic antenna effect, namely photosensitized energy transfer (PSET) from ligand to Ln^3+^ [46, 47]. Ln^3+^ captured triplet energy from BOP@BA via this PSET pathway, then decayed to the ground state via characteristic f–f transitions and nonradiative decay. This established a competing deactivation channel against the intrinsic radiative decay of BOP@BA triplet excitons. The uncoordinated BOP@BA units within the composite retained long‐lived BRTP emission. Consequently, BOP@BA‐Ln exhibited distinct multicolor luminescence under UV illumination and persistent blue afterglow upon cessation of UV excitation.

**FIGURE 6:**
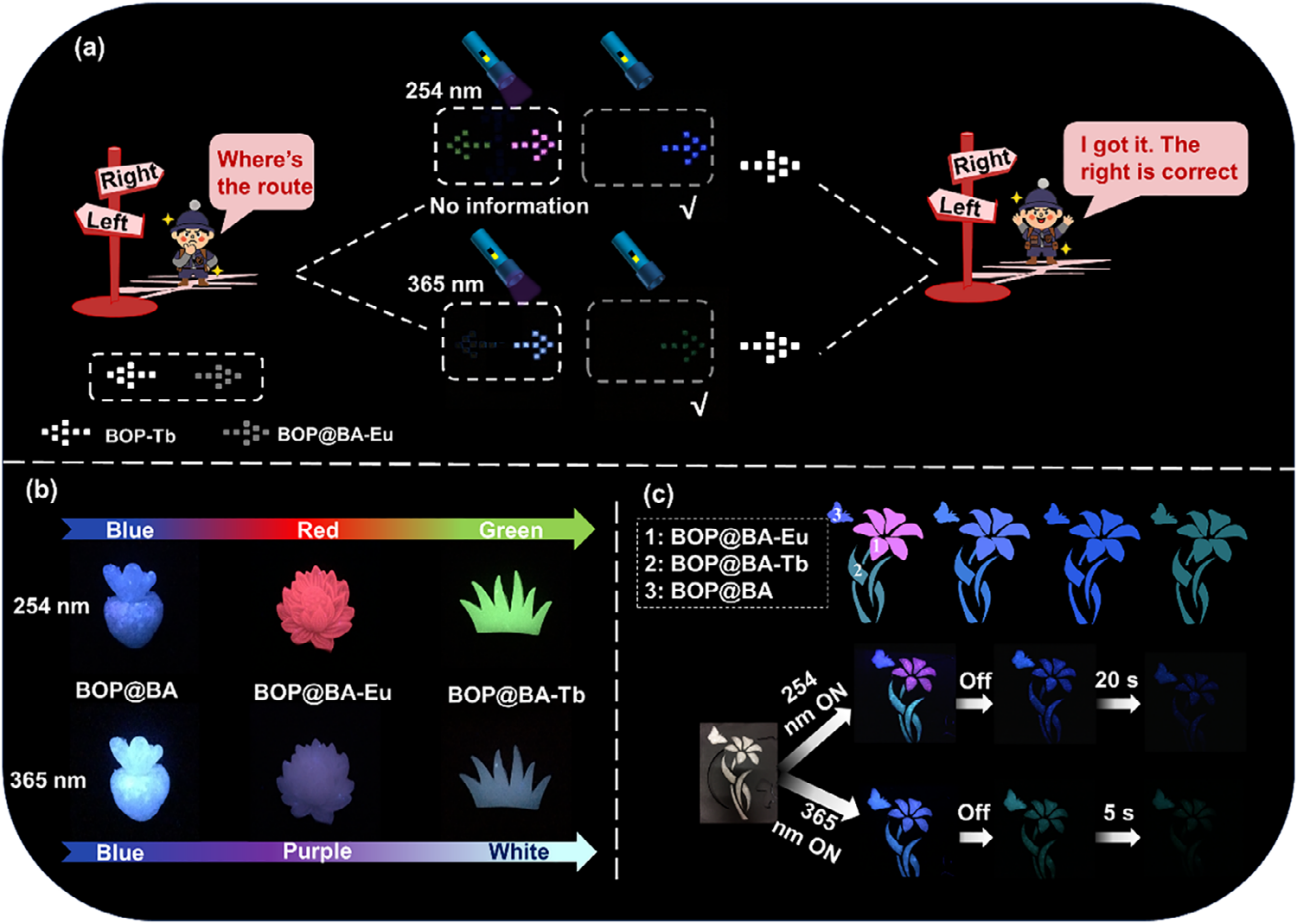
(a) Encrypted road signs controlled by UV light; (b) Multicolor luminescence of BOP@BA adjusted by Eu^3+^ and Tb^3+^ under 254 nm or 365 nm UV light; (c) An encrypted butterfly hidden in a colorful flower above 20 s based on blue afterglow.

By virtue of distinct fluorescence/phosphorescence color contrast, excitation‐dependent emission characteristics, tunable phosphorescence lifetime, and ultralong afterglow, diverse information encryption and anti‐counterfeiting prototypes have been developed. As illustrated in Figure 6a, an encryption indicator displays a green and pink arrow under 254 and 365 nm UV irradiation, respectively, arising from the characteristic emissions of Tb^3+^ (BOP@BA‑Tb^3+^) and Eu^3+^ (BOP@BA‑Eu^3+^). Under UV excitation, these optical outputs deliver misleading information suggesting that “both paths lead to the destination”. In sharp contrast, the correct encrypted information can be deciphered only after switching off the UV light source. Conceptually, this encryption design is analogous to a philosophical metaphor: true insight and truth can only be attained after eliminating external distractions.

Leveraging the excitation‐dependent luminescence of BOP@BA and the characteristic Ln^3+^ emission, multicolor luminescence was achieved by varying Ln^3+^ dopant species and adjusting excitation wavelength (Figure 6b). As depicted in Figure 6c, a vivid pattern consisting of a butterfly and a flower was assembled from different composite materials (butterfly: BOP@BA, lotus: BOP@BA‐Eu, stems: BOP@BA‐Tb). Under alternating UV irradiation, this integrated architecture presented a full‐color luminescent display with distinguishable chromatic outputs. By employing the optical response, luminescent color, and afterglow duration as triple encryption keys, the critical encrypted information (the blue butterfly) gradually emerged with high contrast and clarity within 20 s after switching off the 254 nm UV excitation. Although the emission color varied after Ln^3+^ introduction, color interference disappeared when the UV lamp was off, and regulated blue afterglow could still be obtained from BOP@BA‐Ln by adjusting Ln^3+^ dopants. To the best of our knowledge, phosphorescent materials are widely employed for information encryption owing to their long lifetimes and persistent afterglow, and variable phosphorescence lifetimes are closely correlated with multistage security classification. Therefore, this study not only provides deep insights into the precise regulation of triplet exciton radiative lifetimes but also develops a viable strategy to obtain diverse ultralong blue phosphorescence for multilevel information encryption.

**FIGURE 7:**
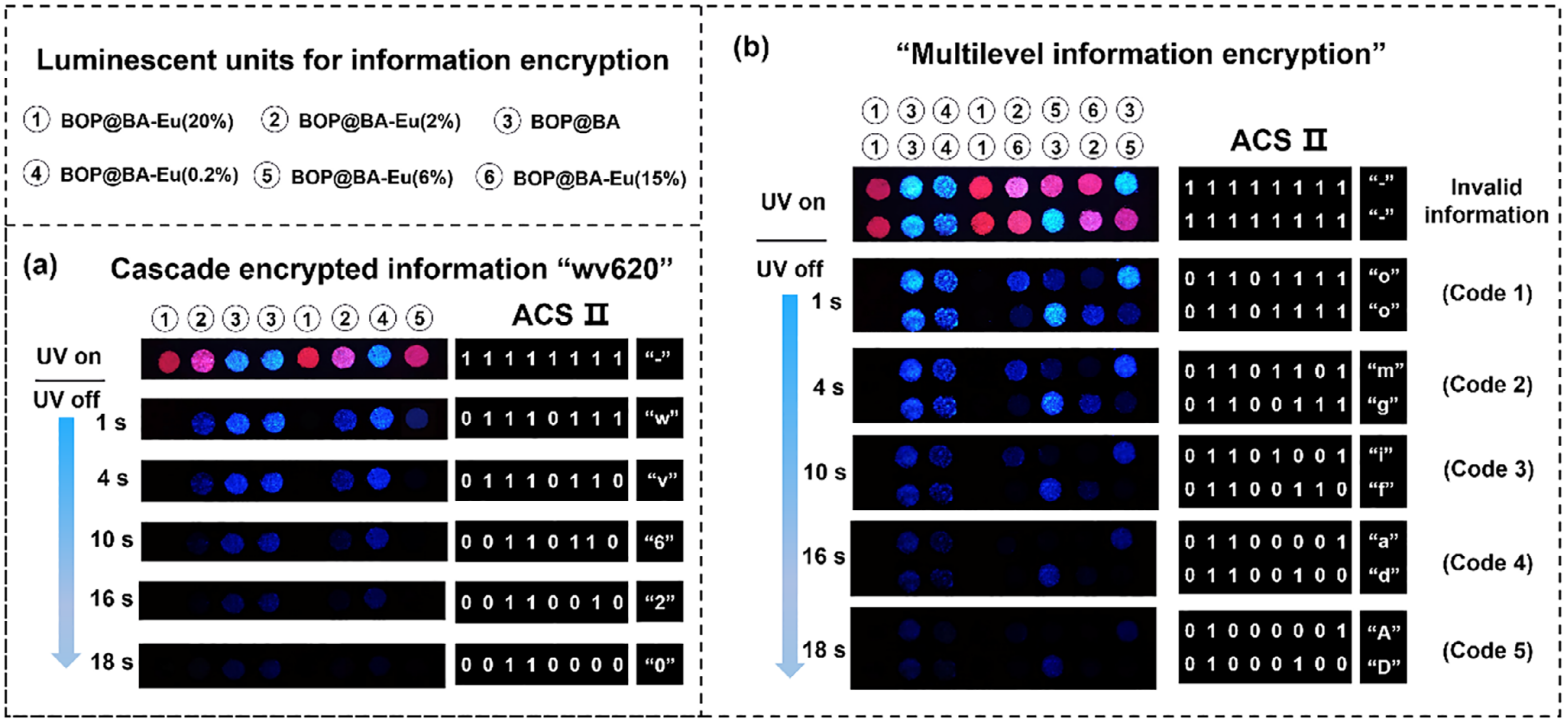
Time‐dependent (a) cascade information encryption and (b) multilevel information encryption (excitation wavelength: 254 nm).

To illustrate this practical significance, time‐resolved cascade and multilevel information encryption prototypes were fabricated, guided by distinct phosphorescence lifetimes and their corresponding afterglow signals (Figure 7). Two dot matrices (8 × 1 and 8 × 2) were constructed to demonstrate cascade‐encrypted information and multilevel encryption, respectively. As shown in Figure 7a, misleading information emerged under 254 nm UV excitation due to the interference of multicolor luminescence. After UV irradiation was terminated, discrete dots of the 8 × 1 matrix decayed at markedly different rates within 18 s, and segmented hidden information emerged sequentially. With ASC II (American Standard Code for Information Interchange) adopted as the decoding rule, discrete fragments “w”, “v”, “6”, “2”, and “0” were extracted in sequence after switching off the UV light for 1, 4, 10, 16, and 18 s. Assembling these sequential fragments, the full ciphertext “wv620” was composed of letters and numerals, thus achieving a time‐dependent cascade information encryption. Conversely, the 8 × 2 dot matrix was designed to embed independent information accessible at various post‐UV delay times (Figure 7b). Decoding the matrix at five fixed time points (1, 4, 10, 16, and 18 s) sequentially produced “oo”, “mg” (mass unit), “if” (a word of conjunction), “ad” (abbreviation for advertisement), and “AD”. Since each delay time corresponded to an independent decryption target, the difficulties of counterfeit identification were substantially improved. We selected the cipher “AD” obtained at the last moment (18 s) as the designated information to highlight “Acceptor‐Donor” in the photosensitized energy transfer.

## 3 Conclusion

In summary, a pure deep BRTP material has been successfully prepared via a direct dehydration reaction between BA and BOP under high‐temperature calcination, with no additional post‐treatment required. The dehydration process not only constructs a rigid covalent crosslinked skeleton that effectively suppresses nonradiative deactivation pathways, including molecular motion and quenching by impurities, but also gives rise to an emission blueshift to 415 nm relative to the individual BA and BOP precursors, thereby delivering ultralong pure deep BRTP with a lifetime up to 2.45 s. Using Ln^3+^ (Eu^3+^ and Tb^3+^) as a “precision modulator”, the phosphorescence lifetime can be finely regulated via supramolecular coordination and subsequent energy transfer, achieving a control precision below 5% and a tunable lifetime window from 2.45 s down to 1.71 s. By exploiting the distinct features of luminescence color, lifetime, and afterglow, multifunctional information encryption and anti‐counterfeiting platforms have been successfully constructed. This work thus provides a reliable and versatile strategy for developing long‐lived pure deep BRTP materials and for achieving their precise luminescence manipulation.

## Author Contributions

**Xingting Que**: data curation, investigation, validation, visualization. **Wen‐Wen Xu**: writing – original draft, data curation, validation, visualization. **Jun Wang**: conceptualization, methodology, supervision, funding acquisition, writing – review and editing. **Wei‐Lei Zhou**: funding acquisition, validation, resources. **Zhiming Chen**: funding acquisition, formal analysis. **Jingsheng Zhang**: software.

## Conflicts of Interest

The authors declare no conflicts of interest.

## Supporting information




**Supporting File**: advs77398‐sup‐0001‐SuppMat.docx.

## Data Availability

The data that support the findings of this study are available from the corresponding author upon reasonable request.

## References

[1] J. Zhang , S. Xu , L. Zhang , et al., “Highly Efficient and Robust Full‐Color Organic Afterglow Through 2D Superlattices Embedment,” Advanced Materials 34 (2022): 2206712, 10.1002/adma.202206712. 36086873

[2] S. Chen , Y. Liu , Y. Zhang , et al., “Ultralong Room‐Temperature Phosphorescence Achieved by Microcapsule Rupture‐Triggered In‐Situ Polymerization for High‐Contrast Damage Visualization and Advanced Anti‐Counterfeiting,” Advanced Materials 38 (2026): 14584, 10.1002/adma.202514584. 41243588

[3] H. Ding , Z. Wang , Z. Ma , et al., “Full‐Color Tunable Time‐Dependent Room‐Temperature Phosphorescence From Self‐Protective Carbonized Polymer Dots,” Advanced Materials 37 (2025): 2418722, 10.1002/adma.202418722. 40223471

[4] D. Wang , J. Gong , Y. Xiong , et al., “Achieving Color‐Tunable and Time‐Dependent Organic Long Persistent Luminescence via Phosphorescence Energy Transfer for Advanced Anti‐Counterfeiting,” Advanced Functional Materials 33 (2023): 2208895, 10.1002/adfm.202208895.

[5] W. Yao , C. Mei , H. Xing , et al., “Polymers Microspheres as Colorful Ultralong Organic Phosphorescence Delivery for Printable Optical Multiplexing,” Advanced Materials 37 (2025): 2502169, 10.1002/adma.202502169. 40556574

[6] Q. Zhang , S. Xu , L. Zhang , L. Yang , and C. Jiang , “Multiemitting Ultralong Phosphorescent Carbonized Polymer Dots via Synergistic Enhancement Structure Design,” Advanced Science 11 (2024): 2400781, 10.1002/advs.202400781. 38552147 PMC11095232

[7] Z. Zhang , Z. Wang , X. Liu , Y.‐E. Shi , Z. Li , and Y. Zhao , “Modulating Emission of Boric Acid Into Highly Efficient and Color‐Tunable Afterglow via Dehydration‐Induced Through‐Space Conjugation,” Advanced Science 10 (2023): 2300139, 10.1002/advs.202300139. 36950728 PMC10214226

[8] M. W. Ha , C. W. Joo , J. Park , et al., “Ancillary Ligand Effect With Methyl and T‐Butyl for Deep Blue and High EQE Blue Phosphorescent Organic Light‐Emitting Diodes,” Chemical Engineering Journal 411 (2021): 128437, 10.1016/j.cej.2021.128437.

[9] Y. Hyun , S. Han , D. Lee , J. Sun , C. Chu , and J. Y. Lee , “Dibenzooxasiline‐Derived n ‐Type Host for High Efficiency and Long‐Lifetime Blue Phosphorescent Organic Light‐Emitting Diodes,” Advanced Optical Materials 13 (2025): 2403436, 10.1002/adom.202403436.

[10] Y. S. Kim , J. Lim , J. Y. Lee , Y. Lee , and C. Choo , “Benzonitirile Modified N Type Host for Exciplex Host to Enhance Efficiency and Lifetime in Blue Phosphorescent Organic Light‐emitting Diodes,” Chemical Engineering Journal 429 (2022): 132584, 10.1016/j.cej.2021.132584.

[11] J. H. Yun , K. H. Lee , W. J. Chung , J. Y. Lee , Y. Lee , and J. J. Lyu , “Thermally Activated Delayed Fluorescence Type Exciplex Host for Long Lifetime in Deep Blue Phosphorescent Organic Light‐emitting Diodes,” Chemical Engineering Journal 417 (2021): 128086, 10.1016/j.cej.2020.128086.

[12] Y.‐J. Yang , D. Ari , Z.‐H. Yu , et al., “Pure Hydrocarbon Hosts Enabling Efficient Multi‐Resonance TADF Blue‐Emitting Organic Light‐Emitting Diodes,” Angewandte Chemie International Edition 64 (2025): 202501895, 10.1002/anie.202501895. PMC12070359 40008831

[13] L. Xiao , Z. Chen , B. Qu , et al., “Recent Progresses on Materials for Electrophosphorescent Organic Light‐Emitting Devices,” Advanced Materials 23 (2011): 926–952, 10.1002/adma.201003128. 21031450

[14] Y. Ding , X. Wang , M. Tang , and H. Qiu , “Tailored Fabrication of Carbon Dot Composites With Full‐Color Ultralong Room‐Temperature Phosphorescence for Multidimensional Encryption,” Advanced Science 9 (2022): 2103833, 10.1002/advs.202103833. 34799998 PMC8787396

[15] X. Han , H.‐Q. Zheng , Y. Yang , Y. Cui , and G. Qian , “Tunable Room‐Temperature Phosphorescence in Hydrogen‐Bonded Organic Crystals via H‐Bonding Units,” Advanced Functional Materials 35 (2025): 2425934, 10.1002/adfm.202425934.

[16] H. Peng , G. Xie , Y. Cao , et al., “On‐demand Modulating Afterglow Color of Water‐soluble Polymers through Phosphorescence FRET for Multicolor Security Printing,” Science Advances 8 (2022): abk2925, 10.1126/sciadv.abk2925. PMC9012460 35427159

[17] M. Shi , Q. Gao , M. Chen , et al., “Hierarchical Luminescence Center Coupling Enables Time‐Dependent Phosphorescence Color From Self‐protective Carbonized Polymer Dots,” Nature Communications 16 (2025): 7473, 10.1038/s41467-025-62807-6. PMC12343937 40796781

[18] H. Yang , Y. Wang , X. Yao , et al., “Efficient and Ultralong Room Temperature Phosphorescence From Isolated Molecules Under Visible Light Excitation,” Journal of the American Chemical Society 147 (2025): 1474–1481, 10.1021/jacs.4c08889. 39653382

[19] J. You , R. Tian , C. Yin , J. Wang , J. Zhang , and J. Zhang , “Organic Circularly Polarized Room‐Temperature Phosphorescence Toolbox With Excellent Practicality and Functionality,” ACS Nano 19 (2025): 38219–38230, 10.1021/acsnano.5c03923. 41146404

[20] C. Chen , X. Zhang , Z. Gao , G. Feng , and D. Ding , “Preparation of AIEgen‐Based near‐Infrared Afterglow Luminescence Nanoprobes for Tumor Imaging and Image‐Guided Tumor Resection,” Nature Protocols 19 (2024): 2408–2434, 10.1038/s41596-024-00990-4. 38637702

[21] M. Morita , S. Yamada , M. Yasui , and T. Konno , “Halogen Bonding as a Supramolecular Strategy for Tailoring Organic Phosphorescence,” Coordination Chemistry Reviews 553 (2026): 217552, 10.1016/j.ccr.2025.217552.

[22] J. You , X. Zhang , Q. Nan , et al., “Aggregation‐Regulated Room‐Temperature Phosphorescence Materials With Multi‐Mode Emission, Adjustable Excitation‐Dependence and Visible‐Light Excitation,” Nature Communications 14 (2023): 4163, 10.1038/s41467-023-39767-w. PMC10344924 37443312

[23] X. Dou , X. Wang , X. Xie , J. Zhang , Y. Li , and B. Tang , “Advances in Polymer‐Based Organic Room‐Temperature Phosphorescence Materials,” Advanced Functional Materials 34 (2024): 2314069, 10.1002/adfm.202314069.

[24] H. Qiu , Y. Yan , H. Zhou , et al., “Stimuli‐Responsive Metal‐Free Phosphorescent Materials: From Design And Mechanisms To Emerging Applications,” Coordination Chemistry Reviews 538 (2025): 216734, 10.1016/j.ccr.2025.216734.

[25] G. Yin , J. Zhou , W. Lu , et al., “Targeting Compact and Ordered Emitters by Supramolecular Dynamic Interactions for High‐performance Organic Ambient Phosphorescence,” Advanced Materials 36 (2024): 2311347, 10.1002/adma.202311347. 38335472

[26] Z. Yin , Z. Wu , and B. Liu , “Recent Advances in Impurity‐Induced Room‐Temperature Phosphorescence,” Advanced Materials 37 (2025): 2506549, 10.1002/adma.202506549. 40465363

[27] S. Feng , Y. Ma , S. Wang , et al., “Light/Force‐Sensitive 0D Lead‐Free Perovskites: From Highly Efficient Blue Afterglow to White Phosphorescence With Near‐Unity Quantum Efficiency,” Angewandte Chemie International Edition 61 (2022): 202116511, 10.1002/anie.202116511. 35015323

[28] J.‐S. Huh , M. J. Sung , S.‐K. Kwon , Y.‐H. Kim , and J.‐J. Kim , “Highly Efficient Deep Blue Phosphorescent OLEDs Based on Tetradentate Pt(II) Complexes Containing Adamantyl Spacer Groups,” Advanced Functional Materials 31 (2021): 2100967, 10.1002/adfm.202100967.

[29] H. Liu , W. Ye , Y. Mu , et al., “Highly Efficient Blue Phosphorescence From Pillar‐Layer MOFs by Ligand Functionalization,” Advanced Materials 34 (2022): 2107612, 10.1002/adma.202107612. 34806790

[30] H.‐Y. Park , A. Maheshwaran , C.‐K. Moon , et al., “External Quantum Efficiency Exceeding 24% With CIE y Value of 0.08 using a Novel Carbene‐Based Iridium Complex in Deep‐Blue Phosphorescent Organic Light‐Emitting Diodes,” Advanced Materials 32 (2020): 2002120, 10.1002/adma.202002120. 32519386

[31] S. N. T. Phan , N. B. Nguyen , and T. S. Teets , “Recent Advances in Using Strong‐Field Ancillary Ligands to Support Blue‐Emitting Iridium(III) and Platinum (II) Complexes,” Chemical Communications 61 (2025): 17544–17558, 10.1039/D5CC02300A. 41122081

[32] Y. H. Nguyen , Y. Wu , V. Q. Dang , C. Jiang , and T. S. Teets , “Combined Nucleophilic and Electrophilic Functionalization to Optimize Blue Phosphorescence in Cyclometalated Platinum Complexes,” Journal of the American Chemical Society 146 (2024): 9224–9229, 10.1021/jacs.4c00203. 38517326

[33] Y. Shoji , Y. Ikabata , I. Ryzhii , et al., “An Element‐Substituted Cyclobutadiene Exhibiting High‐Energy Blue Phosphorescence,” Angewandte Chemie International Edition 60 (2021): 21817–21823, 10.1002/anie.202106490. 34097333

[34] L. Zhou , J. Song , Z. He , et al., “Achieving Efficient Dark Blue Room‐Temperature Phosphorescence With Ultra‐Wide Range Tunable‐Lifetime,” Angewandte Chemie International Edition 63 (2024): 202403773, 10.1002/anie.202403773. 38527962

[35] C. Li , M. Wu , Z. Zhao , J. W. Y. Lam , B. Xu , and B. Z. Tang , “Suppressing Molecular Motions: A Pathway to Enhanced Organic Room‐Temperature Phosphorescence,” Chemistry 11 (2025): 102654, 10.1016/j.chempr.2025.102654.

[36] S. Cai , Z. Sun , H. Wang , et al., “Ultralong Organic Phosphorescent Foams With High Mechanical Strength,” Journal of the American Chemical Society 143 (2021): 16256–16263, 10.1021/jacs.1c07674. 34550674

[37] S. Kong , H. Wang , J. Liao , Y. Xiao , T. Yu , and W. Huang , “Intrinsic Narrowband Blue Phosphorescent Materials and Their Applications in 3D Printed Self‐monitoring Microfluidic Chips,” Advanced Materials 36 (2024): 2412468, 10.1002/adma.202412468. 39422041

[38] K. Ling , S. Li , Y. Wang , et al., “Highly Efficient and Ultralong Organic Phosphorescence by Doping Crown Ether Derivatives Into Polymer,” Chinese Chemical Letters 37 (2025): 112110, 10.1016/j.cclet.2025.112110.

[39] H. Thomas , D. L. Pastoetter , M. Gmelch , et al., “Aromatic Phosphonates: A Novel Group of Emitters Showing Blue Ultralong Room Temperature Phosphorescence,” Advanced Materials 32 (2020): 2000880, 10.1002/adma.202000880. 32239561

[40] S. Zong , B. Wang , J. Zhang , et al., “Confinement Microenvironment Regulation of Carbon Dots in Zeolite for Multi‐Mode Time‐Dependent Phosphorescence Color Evolution,” Angewandte Chemie International Edition 64 (2025): 202420156, 10.1002/anie.202420156. 39559906

[41] W. Ye , H. Ma , H. Shi , et al., “Confining Isolated Chromophores for Highly Efficient Blue Phosphorescence,” Nature Materials 20 (2021): 1539–1544, 10.1038/s41563-021-01073-5. 34426660

[42] M. Wu , Y. Guan , P. Wang , et al., “High‐Temperature‐Available Organic Blue Phosphorescence for Optical Waveguide,” Advanced Functional Materials 35 (2025): 2505113, 10.1002/adfm.202505113.

[43] J. Sun , H. Ahn , S. Kang , et al., “Exceptionally Stable Blue Phosphorescent Organic Light‐Emitting Diodes,” Nature Photonics 16 (2022): 212–218, 10.1038/s41566-022-00958-4.

[44] M. J. Frisch , G. W. Trucks , and H. B. Schlegel , Gaussian 03 (Gaussian, Inc., 2004).

[45] X.‐Y. Dai , M. Huo , and Y. Liu , “Phosphorescence Resonance Energy Transfer From Purely Organic Supramolecular Assembly,” Nature Reviews Chemistry 7 (2023): 854–874, 10.1038/s41570-023-00555-1. 37993737

[46] L. Li , J. Zhou , J. Han , et al., “Finely Manipulating Room Temperature Phosphorescence by Dynamic Lanthanide Coordination toward Multi‐Level Information Security,” Nature Communications 15 (2024): 3846, 10.1038/s41467-024-47674-x. PMC11078970 38719819

[47] F.‐J. Song , P. Su , X. Li , C.‐H. Yan , J.‐L. Zhang , and Y. Tang , “Harnessing Rare Earth Coordination Chemistry for Advanced Photofunctional Materials: From Fundamental Principles to Emerging Technologies,” Chemical Society Reviews 55 (2026): 5117–5197, 10.1039/D5CS00384A. 42052691

