# Supplementary material for: Precisely Regulating 2.45 s Pure Blue Room‐Temperature Phosphorescence via Supramolecular Coordination
Source: Adv Sci (Weinh). 2026 Aug 24:e77398. Online ahead of print. doi: 10.1002/advs.77398 (PMC13502342; doi:10.1002/advs.77398)
Supplement: Supplementary file 1 [file ADVS-9999-e77398-s001.docx]

Supporting Information

Precisely Regulating 2.45 s Pure Blue Room-Temperature Phosphorescence via Supramolecular Coordination

Jun Wang*^a,^ ^§^, Xingting Que^a, §^, Zhiming Chen^a^, Jingsheng Zhang^a^, Wei-Lei Zhou*^b^, Wen-Wen Xu*^c^

J. Wang, X. Que, Z. Chen, J. Zhang

[a] School of Chemistry and Materials Science, Guizhou Normal University, Guiyang 550001, P. R. China
E-mail: wjc@gznu.edu.cn

W.-L. Zhou

[b] Inner Mongolia Key Laboratory for the Natural Products Chemistry and Functional Molecular Synthesis, The Technological Innovation Center of Supramolecular Chinese (Mongolian) Medicine, College of Chemistry and Material Science, Inner Mongolia Minzu University, Tongliao 028000, P. R. China

E-mail: zhouweilei@imun.edu.cn

W.-W. Xu
[c] Department of Thoracic Surgery, Xinqiao Hospital, Army Medical University (Third Military Medical University), Chongqing 400037, P. R. China

E-mail: wenwenxu@tmmu.edu.cn

[§]	J. Wang and X. Que contributed equally to this work

**Section Ⅰ. Materials/Methods/Instrumentation**

All reagents and solvents were commercially available and used without further purification, unless otherwise noted. Boric acid (BA, ≥99.5%, AR) was purchased from Macklin (Shanghai, China). 3,5-dicarboxyphenylboronic acid (BOP, 98%) was obtained from Adamas-beta (Shanghai, China). Rare earth nitrate salts, including Eu(NO_3_)_3_·6H_2_O and Tb(NO_3_)_3_·6H_2_O (purity: 99.99 %), were purchased from Innochem (Beijing, China). Unless otherwise stated, all commercial chemicals and solvents were used without further purification.

X-ray photoelectron spectroscopy (XPS) was carried out with a Thermo Scientific K-Alpha (Manchester, UK). Fourier transform infrared (FTIR) spectra were measured using a Thermo Scientific Nicolet 6700 FT-IR. Scanning electron microscopy (SEM) images were acquired on a ZEISS Sigma 360 microscope. N_2_ adsorption–desorption isotherms were recorded at 77 K using a MicrotracBEL-BELSORP MINI X instrument (Japan). Thermogravimetric analysis (TGA) was performed on a METTLER TOLEDO TGA2 system. X-ray powder diffraction (XRD) patterns were recorded on a Malvern Panalytical X’Pert3 Powder diffractometer. Field-emission transmission electron microscopy (TEM) images were obtained on an HT7700 instrument. Phosphorescence spectra and lifetimes were measured using FL4600 and FluoroMax⁺ spectrometers. The temperature-dependent emission spectra at 77 K were recorded on an Edinburgh FS5 instrument. Steady-state fluorescence spectra and luminescence lifetimes were collected on FL9700 and FLS1000 fluorometers. Luminescence images and afterglow photographs were captured using an iPhone 5 (A1530) at room temperature under a UV lamp.

**Section Ⅱ. Summarizing recent developments of blue RTP materials.**

| **No.** | **Name** | **Phos. emissions** | **Phos. lifetimes** | **Ref.** |
| --- | --- | --- | --- | --- |
| 1 | MCATMA | 408 nm | 1300 ms | [1] |
| 2 | ICz@PU | 465 nm | 1524 ms | [2] |
| 3 | 5,11-ICz/5,7-ICz/5,12-ICz/11,12-ICz@PVA | 440/419/401/429 nm | 0.38/1.50/1.54/2.04 s | [3] |
| 4 | PAM-CzA | 450 nm | 3680 ms | [4] |
| 5 | BA@PHA/BA@MHA/BA@OHA | 420/437/400 nm | 2.01/1.85/0.73 s | [5] |
| 6 | HOC-PM-DMSO | 446 nm | 121 ms | [6] |
| 7 | PAMCz | 414 nm | 4.2 s | [7] |
| 8 | X-CPDs_-3_ | 438 nm | 147 ms | [8] |
| 9 | PHA in PVA | 468 nm | 2.22 s | [9] |
| 10 | CEC/BI | 499 nm | 20 ms | [10] |
| 11 | PA6InCl9 | 466 nm | 1.2 s | [11] |
| 12 | TMA/TDMA/TDEA | 411 nm | 163.7/186.2/169.7 ms | [12] |
| 13 | P-NHCH_3_ | 445 nm | 1925.8 ms | [13] |
| 14 | Hyp-Pro-Gly@PVA | 434 nm | 1240 ms | [14] |
| 15 | 2O-Cphpz@HEA-AA | 448 nm | 1080 ms | [15] |
| 16 | BDPB-OH | 454, 487, 518 nm | 1140 ms | [16] |
| 17 | CDs@MnAPO-5 | 440 nm | 53.1 ms | [17] |
| 18 | TSP | 447 nm | 168.39 ms | [18] |
| 19 | 4TPYBOH@PVA | 450, 480 nm | 0.629 s | [19] |
| 20 | PVA/PMACa-DS | 460 nm | 13.4 ms | [20] |
| 21 | DB/CBBU | 445 nm | 3380 ms | [21] |
| 22 | pP2CA/pPCAA/pP2Am@Mg(H_2_PO_4_)_2_ | 427/425/424 nm | 0.84/0.96/1.26 s | [22] |
| 23 | TAD-HPCD/PVA | 430 nm | 102.99 ms | [23] |
| 24 | B-CDs@SiO_2_ | 447 nm | 1.41 s | [24] |
| 25 | TP@PVA-0.10% | 465 nm | 5.92 s | [25] |
| 26 | Col/PVA/B | 436 nm | 0.88 s | [26] |
| 27 | ODIA/DIA@PMMA | 454/449 nm | 2133/77 ms | [27] |
| 28 | MSNs-CPDs | 430 nm | 176.9 ms | [28] |
| 29 | P2BA_0.1%_@U60 | 416 nm | 5.33 s | [29] |
| 30 | 2J5X/PVA | 446 nm | 2.516 s | [30] |
| 31 | PAA-CPBA | 432 nm | 523 ms | [31] |
| 32 | TPP-2C-Cz | 445 nm | 1291.32 ms | [32] |
| 33 | BClPPBr-PVA-2.0% | 480 nm | 76 ms | [33] |
| 34 | Poly-An-CD | 459 nm | 0.181 ms | [34] |
| 35 | Glycolylurea | 458 nm | 1.24 s | [35] |
| 36 | pCBP | 430 nm | 52.1 ms | [36] |
| 37 | B–CDs@PAM | 476 nm | 637 ms | [37] |
| 38 | T-PMMA/T-PC | 480 nm | 4687/2646 ms | [38] |
| 39 | B-CDs@SA | 423 nm | 165.8 ms | [39] |
| 40 | NHPI/NNHPI | 450/480 nm | 527.67/392.54 ms | [40] |
| 41 | m, p-CDs@CA | 416 nm | 1.74 s | [41] |
| 42 | CD-IQ-polymer/CD-IQ/CB[7]-polymer | 450 nm | 238.47/268.9 ms | [42] |
| 43 | N_4_-CsO/N_5_-CsO/N_6_-CsO | 471/438/450 nm | 249.43/0.27/0.74 ms | [43] |
| 44 | P2BA@BA | 412 nm | 8.74 s | [44] |
| 45 | 4N-TCD: DPA: PVP | 420 nm | 522.77 ms | [45] |
| 46 | 2CzB@BA | 435 nm | 5.44 s | [46] |
| 47 | H_2_O-MCA@C1 | 450 nm | 2.3 s | [47] |
| 48 | BA@ICZ | 451 nm | 3.26 s | [48] |
| 49 | CZBPin@PVA | 440 nm | 4.64 s | [49] |
| 50 | 9HFL@PVA | 455 nm | 4.20 s | [50] |
| 51 | IPA@BA | 434 nm | 1.881 s | [51] |
| 52 | MBA | 415 nm | 2.9 s | [52] |
| 53 | ZnAl-LDH[Ph-m(COO^−^)] | 404 nm | 140 ms | [53] |
| 54 | B-CDs | 462 nm | 2.03 s | [54] |
| 55 | SA-CPBA | 432 nm | 231 ms | [55] |
| 56 | 700-CDs@Al_2_O_3_ | 420 nm | 10.59 ms | [56] |

**Section Ⅲ. Preparation of materials.**

**Preparation of DBA, DBOP, and BOP@BA.** DBA, DBOP, and BOP@BA correspond to thermally dehydrated products of neat BA, BOP, and their binary mixture, respectively. BOP@BA was synthesized through a heat-treatment strategy. Typically, 100 mg of boric acid (BA) was dissolved in 10 mL of deionized water in a beaker and mixed with 1~20 mg of 3,5-dicarboxyphenylboronic acid (BOP). Then, the beaker was heated in an oven at 200 °C for 4 h to afford the material BOP@BA. DBA and DBOP were synthesized through a similar procedure.

**Preparation of BOP@BA-Ln^3+^.** Similar to the preparation process of DBA, DBOP, and BOP@BA, the mass ratio of BA to BOP was fixed at 100:5, and varying amounts (0.01-15 mg) of nitrates, Eu(NO_3_)_3_·6H_2_O, and Tb(NO_3_)_3_·6H_2_O, were separately added to the aqueous solution to give the corresponding BOP@BA-Ln materials. All samples were prepared under identical hydrothermal conditions as described above.

**Preparation of BOP+BA.** Solid BOP and BA (mass ratio = 5:100) were co-ground in a mortar with 10 mL of deionized water. The resulting homogeneous slurry was divided into two aliquots and subjected to distinct drying protocols. One aliquot was freeze-dried, while the other was oven-dried at 65 °C under vacuum conditions. Both samples were collected for phosphorescence delay spectral measurements.

**Theoretical calculation.** Ground-state and excited-state optimizations, as well as energy-level calculations, were performed for BOP, BOP@BA, BOP@BA-Eu, and BOP@BA-Tb using Gaussian 2003 software at the PBE0-D3^1^/6-311G(d,p)^2^ + SDD^3^ levels and DFT or TD-DFT methods^[57]^, respectively. The SDD effective core potential was used to describe the atomic orbitals and relativistic effects of the heavy atoms europium and terbium. In addition, the spin-orbit coupling (SOC) constants for BOP and BOP@BA were calculated using the ORCA software package at the PBE0/def2-SV(P) level^[58]^.

**Section Ⅳ. Photophysical properties.**

**Table S1.** Photophysical properties of DBA, DBOP, and BOP@BA, including phosphorescence lifetimes (*τ_Phos_*) and phosphorescence quantum yields (Φ*_Phos_*).

| Samples | Excitation | Fluorescence | Phosphorescence | τ*_Phos_* | Φ*_Phos_* |
| --- | --- | --- | --- | --- | --- |
| DBA | 263 nm | 395 nm | 432 nm | 1.20 s | 7.89% |
| DBOP | 327 nm | 395 nm | 505 nm | 0.07 s | 10.7% |
| BOP@BA | 265 nm | - | 415 nm | 2.45 s | 16.2% |

**Figure S1.** (a) Excitation spectra of solid DBA, DBOP, and BOP@BA under ambient environment; (b) CIE coordinate of BOP@BA.

**Figure S2.** (a) Time-resolved luminescence decay plots and (b) averaged lifetimes of BOP@BA at 415 nm with gradually increasing the mass ratio of BOP.

**Figure S3.** (a) XPS full scan spectra of DBA and BOP@BA; (b) N_2_ adsorption-desorption isotherms and the pore size distribution of BOP@BA (inset).

**Figure S4.** SEM image (a) BOP@BA and (b) DBA.

**Figure S5.** (a) Delay spectra and (b) XRD of BOP@BA under various calcination temperatures.

**Figure S6.** Time-resolved luminescence decay plot of BOP@BA after being exposed to (a) ambient environment (testing humidity stability) and (b) being irradiated by 254 nm UV irradiation (testing light stability) for various durations.

**Figure S7.** Excitation, prompt, and delay spectra of solid BOP under ambient environment.

**Figure S8.** Prompt and delay spectra of dilute BOP solution (2×10⁻^7^ M in 2-methyltetrahydrofuran) at 77 K (excitation wavelength: 330 nm).

**Figure S9.** Delay spectra of BOP+BA mixtures (5 wt%) obtained through freeze-drying treatment and thermal treatment (65 °C), respectively.

**Figure S10.** Temperature-dependent delay spectra of solid BOP@BA with a temperature interval of 10 K (the sudden drop in the peak probably originates from the difference of the testing instrument).

**Figure S11.** FT-IR spectra of BOP@BA after doping Eu^3+^ and Tb^3+^.

**Figure S12.** (a) Delay spectra of BOP@BA, BOP@BA-Eu (BA:BOP:Eu^3+^=100:5:1), and BOP@BA-Tb (BA:BOP:Tb^3+^=100:5:1); (b) Photographs of BOP@BA-Eu (BA:BOP:Eu^3+^ =100:5:1) and BOP@BA-Tb (BA:BOP:Tb^3+^ = 100:5:1) before and after ceasing 254 nm UV light.

**Figure S13.** Prompt spectra of solid (a) BOP@BA-Eu and (b) BOP@BA-Tb with gradually increasing the mass ratio of Eu^3+^and Tb^3+^ under ambient environment, respectively.

**Tables S2.** Phosphorescence lifetimes (*τ_Phos_*), phosphorescence quantum yield (Φ*_Phos_*), and energy transfer efficiencies (ETE) of BOP@BA with increasing Eu^3+^ contents (BOP: 5.0 mg, BA: 100.0 mg).

| Eu^3+^ (mg) | 0.01 | 0.05 | 0.1 | 0.2 | 0.6 | 1 | 1.5 | 2 | 4 | 6 | 10 | 15 |
| --- | --- | --- | --- | --- | --- | --- | --- | --- | --- | --- | --- | --- |
| $\tau_{Phos}$ (s) | 2.31 | 2.21 | 2.17 | 2.08 | 2.05 | 1.97 | 1.95 | 1.91 | 1.88 | 1.82 | 1.79 | 1.71 |
| ETE (%) | 5.71 | 9.8 | 11.43 | 15.1 | 16.33 | 19.59 | 20.41 | 22.04 | 23.27 | 25.71 | 26.94 | 30.2 |
| Φ*_Phos_*  (%) | 14.51 | 11.71 | 8.12 | 5.48 | 5.33 | 2.48 | 1.51 | 1.36 | 0.23 | 0.47 | - | - |

Note: “-” represents that the signals are too low to be detected.

**Tables S3.** *τ_Phos_*, Φ*_Phos_,* and ETE of BOP@BA with increasing Tb^3+^ contents (BOP: 5.0 mg, BA: 100.0 mg).

| Tb^3+^ (mg) | 0.01 | 0.05 | 0.1 | 0.2 | 0.6 | 1.5 | 2 | 4 | 6 | 10 | 15 |
| --- | --- | --- | --- | --- | --- | --- | --- | --- | --- | --- | --- |
| *τ_Phos_*  (s) | 2.3 | 2.28 | 2.17 | 2.11 | 2 | 1.98 | 1.95 | 1.94 | 1.83 | 1.8 | 1.74 |
| ETE (%) | 6.12 | 6.94 | 11.43 | 13.88 | 18.37 | 19.18 | 20.41 | 20.82 | 25.31 | 26.53 | 28.98 |
| Φ*_Phos_*  (%) | 12.84 | 9.71 | 8.11 | 6.03 | 2.18 | 2.09 | 1.66 | 1.59 | 0.7 | - | - |

Note: “-” represents that the signals are too low to be detected.

**Figure S14.** Afterglow photographs of BOP@BA-Eu with a gradually increasing mass ratio of Eu^3+^ before and after ceasing 365 nm UV light.

**Figure S15.** (a) Time-resolved luminescence decay plots and (b) averaged lifetimes of BOP@BA-Tb at 415 nm with gradually increasing the mass ratio of Tb^3+^.

**Figure S16.** Afterglow photographs of BOP@BA-Tb with a gradually increasing mass ratio of Tb^3+^ before and after ceasing 254 nm UV light.

**Figure S17.** Afterglow photographs of BOP@BA-Tb with a gradually increasing mass ratio of Tb^3+^ before and after ceasing 365 nm UV light.

**Figure S18.** DFT optimized geometry with 5.59 Å donor-acceptor separation.

**Figure S19.** Excitation spectra of BOP@BA-Ln with gradually increasing the mass ratio of (a) Eu^3+^ and (b) Tb^3+^ (The marked parts represent charge transfer between ligands and Ln^3+^).

**References**

1 J. Zhang, S. Xu, L. Zhang, et al., "Highly Efficient and Robust Full‐Color Organic Afterglow through 2D Superlattices Embedment," Advanced Materials 34 (2022): 2206712, <https://doi.org/10.1002/adma.202206712>.
2 S. Chen, Y. Liu, Y. Zhang, et al., "Ultralong Room-Temperature Phosphorescence Achieved by Microcapsule Rupture-Triggered In-Situ Polymerization for High-Contrast Damage Visualization and Advanced Anti-counterfeiting," Advanced Materials 38 (2026): e14584, <https://doi.org/10.1002/adma.202514584>.
3 D. Wang, J. Gong, Y. Xiong, et al., "Achieving Color-Tunable and Time-Dependent Organic Long Persistent Luminescence via Phosphorescence Energy Transfer for Advanced Anti-Counterfeiting," Advanced Functional Materials 33 (2023): 2208895, <https://doi.org/10.1002/adfm.202208895>.
4 W. Yao, C. Mei, H. Xing, et al., "Polymers Microspheres as Colorful Ultralong Organic Phosphorescence Delivery for Printable Optical Multiplexing," Advanced Materials 37 (2025): 2502169, <https://doi.org/10.1002/adma.202502169>.
5 Z. Zhang, Z. Wang, X. Liu, et al., "Modulating Emission of Boric Acid into Highly Efficient and Color-Tunable Afterglow via Dehydration-Induced Through-Space Conjugation," Advanced Science 10 (2023): 2300139, <https://doi.org/10.1002/advs.202300139>.
6 X. Han, H.-Q. Zheng, Y. Yang, et al., "Tunable Room-Temperature Phosphorescence in Hydrogen-Bonded Organic Crystals via H-Bonding Units," Advanced Functional Materials 35 (2025): 2425934, <https://doi.org/10.1002/adfm.202425934>.
7 H. Peng, G. Xie, Y. Cao, et al., "On-demand Modulating Afterglow Color of Water-soluble Polymers Through Phosphorescence FRET for Multicolor Security Printing," Science Advances 8 (2022): eabk2925, <https://doi.org/10.1126/sciadv.abk2925>.
8 M. Shi, Q. Gao, M. Chen, et al., "Hierarchical Luminescence Center Coupling Enables Time-dependent Phosphorescence Color from Self-protective Carbonized Polymer Dots," Nature Communications 16 (2025): 7473, <https://doi.org/10.1038/s41467-025-62807-6>.
9 H. Yang, Y. Wang, X. Yao, et al., "Efficient and Ultralong Room Temperature Phosphorescence from Isolated Molecules under Visible Light Excitation," Journal of the American Chemical Society 147 (2025): 1474-1481, <https://doi.org/10.1021/jacs.4c08889>.
10 J. You, R. Tian, C. Yin, et al., "Organic Circularly Polarized Room-Temperature Phosphorescence Toolbox with Excellent Practicality and Functionality," ACS Nano 19 (2025): 38219-38230, <https://doi.org/10.1021/acsnano.5c03923>.
11 S. Feng, Y. Ma, S. Wang, et al., "Light/Force-Sensitive 0D Lead-Free Perovskites: From Highly Efficient Blue Afterglow to White Phosphorescence with Near-Unity Quantum Efficiency," Angewandte Chemie International Edition 61 (2022): e202116511, <https://doi.org/10.1002/anie.202116511>.
12 H. Liu, W. Ye, Y. Mu, et al., "Highly Efficient Blue Phosphorescence from Pillar-Layer MOFs by Ligand Functionalization," Advanced Materials 34 (2022): 2107612, <https://doi.org/10.1002/adma.202107612>.
13 L. Zhou, J. Song, Z. He, et al., "Achieving Efficient Dark Blue Room-Temperature Phosphorescence with Ultra-Wide Range Tunable-Lifetime," Angewandte Chemie International Edition 63 (2024): e202403773, <https://doi.org/10.1002/anie.202403773>.
14 S. Cai, Z. Sun, H. Wang, et al., "Ultralong Organic Phosphorescent Foams with High Mechanical Strength," Journal of the American Chemical Society 143 (2021): 16256-16263, <https://doi.org/10.1021/jacs.1c07674>.
15 S. Kong, H. Wang, J. Liao, et al., "Intrinsic Narrowband Blue Phosphorescent Materials and Their Applications in 3D Printed Self-monitoring Microfluidic Chips," Advanced Materials 36 (2024): 2412468, <https://doi.org/10.1002/adma.202412468>.
16 H. Thomas, D. L. Pastoetter, M. Gmelch, et al., "Aromatic Phosphonates: A Novel Group of Emitters Showing Blue Ultralong Room Temperature Phosphorescence," Advanced Materials 32 (2020): 2000880, <https://doi.org/10.1002/adma.202000880>.
17 S. Zong, B. Wang, J. Zhang, et al., "Confinement Microenvironment Regulation of Carbon Dots in Zeolite for Multi-Mode Time-Dependent Phosphorescence Color Evolution," Angewandte Chemie International Edition 64 (2025): e202420156, <https://doi.org/10.1002/anie.202420156>.
18 W. Ye, H. Ma, H. Shi, et al., "Confining Isolated Chromophores for Highly Efficient Blue Phosphorescence," Nature Materials 20 (2021): 1539-1544, <https://doi.org/10.1038/s41563-021-01073-5>.
19 L. Li, J. Zhou, J. Han, et al., "Finely Manipulating Room Temperature Phosphorescence by Dynamic Lanthanide Coordination Toward Multi-Level Information Security," Nature Communications 15 (2024): 3846, <https://doi.org/10.1038/s41467-024-47674-x>.
20 J. Deng, H. Liu, D. Liu, et al., "Room-Temperature Phosphorescent tough Hydrogels Based on Ionically Crosslinked Nonaromatic Polymers," Advanced Functional Materials 34 (2024): 2308420, https://doi.org/10.1002/adfm.202308420.
   21	Y. Zhou, J. Ma, P. Zhang, et al., "Deep-Blue Ultralong Room-Temperature Phosphorescence from Halogen-Free Organic Materials through Cage Effect for Various Applications," Advanced Optical Materials 9 (2021): 2100959, https://doi.org/10.1002/adom.202100959.
   22	T.-Y. Zhou, Q. Zhao, H.-Y. Zhao, et al., "Organic-Phosphate Hybrids with Colorful Persistent Luminescence," Chemical Engineering Journal 511 (2025): 161969, https://doi.org/10.1016/j.cej.2025.161969.
   23	X. Zhao, X. Zhou, Q. Cheng, et al., "Blue Phosphorescent Solid Supramolecular Assemblies between Hydroxypropyl-β-cyclodextrin and Triazine Derivatives for Achieving Multicolor Delayed Fluorescence," Nano Today 60 (2025): 102561, https://doi.org/10.1016/j.nantod.2024.102561.
   24	J. Liao, X. Chen, J. Li, et al., "Engineering Strategy for Achieving Transparent Aqueous Multicolor Long Lifetime Room Temperature Phosphorescent Carbon Dots," Nano Energy 146 (2025): 111532, https://doi.org/10.1016/j.nanoen.2025.111532.
   25	Q. Wang, X.-L. Xu, M.-Y. Zhu, et al., "Integrating Three-Primary-Color and White Long-lasting Afterglows in Triphenylene-Doped Polymers for Time/Space-Resolved Applications," Chemical Engineering Journal 507 (2025): 160535, https://doi.org/10.1016/j.cej.2025.160535.
   26	H. Jiang, L. Yang, H. Ju, et al., "Ultralong-Lived and Color-Tunable RTP in Biodegradable Natural Fibers Based Textiles for Wearable Security and Display," Small 21 (2025): e11425, https://doi.org/10.1002/smll.202511425.
   27	S. Zhang, G. Liu, Z. Mao, et al., "Organic Dopant Cyclization and Significantly Improved RTP Properties," Chemical Science 15 (2024): 19886-19892, https://doi.org/10.1039/D4SC06213B.
   28	C. Zheng, S. Tao, Y. Liu, et al., "Achieving Blue Water-Dispersed Room-Temperature Phosphorescence of Carbonized Polymer Dots through Nano-Compositing with Mesoporous Silica," Chinese Chemical Letters 33 (2022): 4213-4218, https://doi.org/10.1016/j.cclet.2022.03.041.
   29	Q. Jia, X. Yan, B. Wang, et al., "Construction of Room Temperature Phosphorescent Materials with Ultralong Lifetime by In-Situ Derivation Strategy," Nature Communications 14 (2023): 4164, https://doi.org/10.1038/s41467-023-39795-6.
   30	J. Chen, S. Zhang, G. Liu, et al., "Regulating Organic Dopant Dispersion in Polymer Matrices for Concentration-Controlled Color-Tunable Organic RTP Emissions," Advanced Optical Materials 11 (2023): 2301163, https://doi.org/10.1002/adom.202301163.
   31	L. Kong, Y. Zhu, S. Sun, et al., "Tunable Ultralong Multicolor and Near-Infrared Emission from Polyacrylic Acid-Based Room Temperature Phosphorescence Materials by FRET," Chemical Engineering Journal 469 (2023): 143931, https://doi.org/10.1016/j.cej.2023.143931.
   32	J. Li, G. Xing, J. Wu, et al., "Photoirradiation-Gated Excitation-Dependent Room-Temperature Phosphorescence in Through-Space Charge Transfer Molecules," Laser & Photonics Reviews 18 (2024): 2300704, https://doi.org/10.1002/lpor.202300704.
   33	P. She, J. Duan, F. Li, et al., "Exploring Electronic Coupling Effects in Amorphous Polymers for Multi-Color Persistent Room-Temperature Phosphorescence," Science China Materials 66 (2023): 4749-4755, https://doi.org/10.1007/s40843-023-2651-6.
   34	X. Lin, Q. Xu, X. Ma, "Emission-Tunable Room-Temperature Phosphorescent Polymers Based on Dynamic Reversible Supramolecule-Mediated Photocrosslinking," Advanced Optical Materials 10 (2022): 2101646, https://doi.org/10.1002/adom.202101646.
   35	L. Bian, H. Ma, W. Ye, et al., "Color-Tunable Ultralong Organic Phosphorescence Materials for Visual UV-Light Detection," Science China Chemistry 63 (2020): 1443-1448, https://doi.org/10.1007/s11426-020-9761-x.
   36	J.-X. Wang, Y.-G. Fang, C.-X. Li, et al., "Time-Dependent Afterglow Color in a Single-Component Organic Molecular Crystal," Angewandte Chemie International Edition 132 (2020): 10118-10122, https://doi.org/10.1002/ange.202001141.
   37	H.-y. Wang, L. Zhou, H.-m. Yu, et al., "Exploration of Room-Temperature Phosphorescence and New Mechanism on Carbon Dots in a Polyacrylamide Platform and their Applications for Anti-Counterfeiting and Information Encryption," Advanced Optical Materials 10 (2022): 2200678, https://doi.org/10.1002/adom.202200678.
   38	X. Zheng, Q. Han, Q. Lin, et al., "A Processable, Scalable, and Stable Full-Color Ultralong Afterglow System Based on Heteroatom-Free Hydrocarbon Doped Polymers," Materials Horizons 10 (2023): 197-208, https://doi.org/10.1039/D2MH00998F.
   39	H. Zhang, L. Sun, X. Guo, et al., "Multicolor Fluorescent/Room Temperature Phosphorescent Carbon Dot Composites for Information Encryption and Anti-Counterfeiting," Applied Surface Science 613 (2023): 155945, https://doi.org/10.1016/j.apsusc.2022.155945.
   40	T. Li, Y. Zheng, C. Wu, et al., "Crosslink-Enhanced Strategy to Achieve Multicolor Long-Lived Room Temperature Phosphorescent Films with Excellent Photostability," Chinese Chemical Letters 33 (2022): 4238-4242, https://doi.org/10.1016/j.cclet.2022.03.047.
   41	Y. Zheng, Q. Zhou, Y. Yang, et al., "Full-Color Long-Lived Room Temperature Phosphorescence in Aqueous Environment," Small 18 (2022): 2201223, https://doi.org/10.1002/smll.202201223.
   42	Y. Sun, Y. Chen, L. Jiang, et al., "Purely Organic Blue Room-Temperature Phosphorescence Activated by Acrylamide In-Situ Photopolymerization," Advanced Optical Materials 10 (2022): 2201330, https://doi.org/10.1002/adom.202201330.
   43	Z. Cong, M. Han, Y. Fan, et al., "Ultralong Blue Room-Temperature Phosphorescence by Cycloalkyl Engineering," Materials Chemistry Frontiers 6 (2022): 1606-1614, https://doi.org/10.1039/D2QM00284A.
   44	X. Zheng, Y. Huang, W. Lv, et al., "Nearly Unity Quantum Yield Persistent Room-Temperature Phosphorescence from Heavy Atom-Free Rigid Inorganic/Organic Hybrid Frameworks," Angewandte Chemie International Edition 61 (2022): e202207104, https://doi.org/10.1002/anie.202207104.
   45	X. Zhao, X. He, Y. Gao, et al., "Achieving Efficient Blue Room-Temperature Phosphorescence through Self-Doping of Similar Structures and Polymer Assistance," Advanced Optical Materials 13 (2025): 2402452, https://doi.org/10.1002/adom.202402452.
   46	Z. Guan, Z. Tang, J. Zeng, et al., "Stepwise Stiffening Chromophore Strategy Realizes a Series of Ultralong Blue Room-Temperature Phosphorescent Materials," Advanced Science 11 (2024): 2402632, https://doi.org/10.1002/advs.202402632.
   47	Z.-Y. Chen, Z. Chen, Y.-Q. Zhu, et al., "Ultralong Blue Organic Room-Temperature Phosphorescence Promoted by Green Assembly," Advanced Functional Materials 34 (2024): 2408023, https://doi.org/10.1002/adfm.202408023.
   48	L. Zhou, S. Mu, L. Ma, et al., "Achieving Ultralong Room-Temperature Phosphorescence Via Single-Bond Locking Planarization Strategy," ACS Materials Letters 6 (2024): 5384-5391, https://doi.org/10.1021/acsmaterialslett.4c02116.
   49	D. Ding, X. Xu, F. Li, et al., "High Performance Deep Blue Organic Room Temperature Phosphorescence: 4.64 s Ultra-Long Lifetime and 60.4% Quantum Efficiency Balanced by Simple Phosphor Heteroatom Modulation," Small 21 (2025): e03142, https://doi.org/10.1002/smll.202503142.
   50	Z. Liu, B. Jiang, X. Zhang, et al., "Quasi-Heavy Atom Effect for Room-Temperature Phosphorescence," Materials Horizons 13 (2026): 307-315, https://doi.org/10.1039/D5MH01328C.
   51	Y. Cheng, W. Fan, L. Wang, et al., "Fast Photostimulus-Responsive Ultralong Room-Temperature Phosphorescence Behaviour of Benzoic Acid Derivatives@boric Acid," Journal of Materials Chemistry C 10 (2022): 8806-8814, https://doi.org/10.1039/D2TC00804A.
   52	Z. Zhang, Y.-e. Shi, Y. Liu, et al., "Highly Efficient and Stable Deep-Blue Room Temperature Phosphorescence via Through-Space Conjugation," Chemical Engineering Journal 442 (2022): 136179, https://doi.org/10.1016/j.cej.2022.136179.
   53	Q. Chen, P. Cao, P. Wu, "Enabling Aqueous Phase Long-Lived Deep-Blue Phosphorescence with Layered Double Hydroxide," Advanced Science 12 (2025): 2413896, https://doi.org/10.1002/advs.202413896.
   54	Z. Song, Y. Shang, Q. Lou, et al., "A Molecular Engineering Strategy for Achieving Blue Phosphorescent Carbon Dots with Outstanding Efficiency Above 50%," Advanced Materials 35 (2023): 2207970, https://doi.org/10.1002/adma.202207970.
   55	S. Sun, Y. Zhu, T. Li, et al., "Ultralong Sodium Alginate-Based Room Temperature Phosphorescence Materials with Advantages of Color Tunability, Flexibility and Facile Large-Area Fabrication," Chemical Engineering Journal 485 (2024): 149751, https://doi.org/10.1016/j.cej.2024.149751.
   56	M. Cai, Z. Li, R. Chen, et al., "Room Temperature Phosphorescent Materials Based on Rosin Acid with Time-Dependent Afterglow and Visible Light Excitation," Chemical Engineering Journal 514 (2025): 163281, <https://doi.org/10.1016/j.cej.2025.163281>.

57	M. J. Frisch, G. W. Trucks, H. B. Schlegel, et al., Gaussian 03, Gaussian, Inc., Wallingford, CT, 2004.
58	L. Li, J. Zhou, J. Han, et al., "Finely Manipulating Room Temperature Phosphorescence by Dynamic Lanthanide Coordination Toward Multi-Level Information Security," *Nature Communications* 15 (2024): 3846, <https://doi.org/10.1038/s41467-024-47674-x>.
